# Banana breeding by genome design

**DOI:** 10.1111/jipb.70025

**Published:** 2025-09-11

**Authors:** Rida Arshad, Tayyaba Razzaq, Bilal Ahmad, Ting Hou, Chaochao Li, Zhongxin Jin, Wei Zhang, Zhongjie Liu, Hui‐Run Huang, Peitao Lü, Wei Wang, Xue‐Jun Ge, Yongfeng Zhou, Jianghui Xie

**Affiliations:** ^1^ State Key Laboratory of Tropical Crop Breeding, Shenzhen Branch, Guangdong Laboratory of Lingnan Modern Agriculture, Key Laboratory of Synthetic Biology, Ministry of Agriculture and Rural Affairs, Agricultural Genomics Institute at Shenzhen Chinese Academy of Agricultural Sciences Shenzhen 518000 China; ^2^ State key Laboratory of Tropical Crop Breeding, Institute of Tropical Bioscience and Biotechnology & Sanya Research Institute Chinese Academy of Tropical Agricultural Sciences Haikou 571101 China; ^3^ Key Laboratory of National Forestry and Grassland Administration on Plant Conservation and Utilization in Southern China, South China Botanical Garden the Chinese Academy of Sciences Guangzhou 510650 China; ^4^ State Key Laboratory of Tropical Crop Breeding, Tropical Crops Genetic Resources Institute & Sanya Research Institute Chinese Academy of Tropical Agricultural Sciences Haikou 571101 China

**Keywords:** bananas, domestication, genetics, genomic breeding, genomics, Musa

## Abstract

Bananas and plantains of the genus *Musa* constitute the most vital fruits and staple foods. Cultivated bananas may have originated from intraspecific and interspecific hybridizations of four wild species, namely *Musa acuminata* (A), *M. balbisiana* (B), *M. schizocarpa* (S), and the *Australimusa* species (T). Here, we appraise the advances made in banana genomics, genetics, and breeding over the past few decades. The sequencing of *Musa* genomes has been a major breakthrough in banana research programs, presenting unprecedented possibilities for gaining deeper insights into the evolution, domestication, breeding, and genetics of indispensable agronomic traits of bananas. Also, we delve into how these genetic facets, coupled with innovative genomic‐assisted tools, including genomic selection and gene editing, propel advancements in banana breeding endeavors. Ultimately, we propose the forthcoming prospects within the domain of banana genetics and breeding.

## Graphical abstract

Bananas and plantains likely originated from hybridizations of four wild *Musa* species. This review highlights advances in banana genomics, genetics, and breeding, emphasizing genome sequencing breakthroughs and genomic‐assisted tools like selection and gene editing, and explores future prospects for improving key agronomic traits.

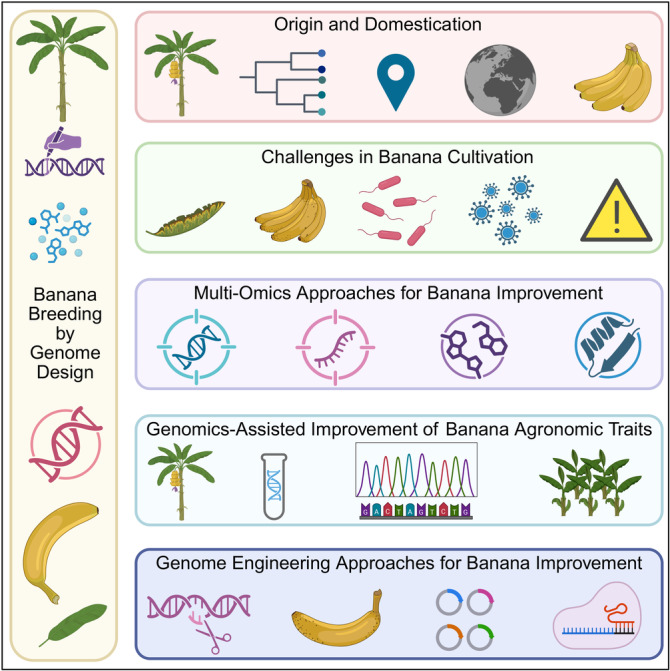

## INTRODUCTION

Bananas—inclusive of plantains—are regarded as one of the globally pivotal fresh fruits and serve as a primary staple food for a weighty population in developing countries. They are consumed in many diverse ways, such as raw, cooked, baked, steamed, or even fermented (Fungo and Pillay, 2011). Bananas hold substantial importance globally, with most of their production stemming from small‐scale farmers who either consume them locally or engage in domestic trade. They seize the fourth spot among the mainstay food crops in underdeveloped countries—as classified by the United Nations—based on overall production and food consumption (Rouard et al., 2022). Annually, 179.3 million tons of bananas are produced worldwide, and the total value of banana exports amounts to an impressive US$13.8 billion, accounting for a volume of 26.48 million tons (FAOSTAT, 2022).

Bananas and plantains belong to an extensive genus *Musa* of the Musaceae family. Musaceae entails three genera (*Musa*, *Ensete*, and the monospecific *Musella*), where *Musa* stands out as the most diverse, covering over 75 defined species (spp.) and subspecies (ssp.) (Häkkinen, 2013; Mertens et al., 2021a; Kallow et al., 2022). Domesticated bananas were derived through interspecific or intraspecific hybridization between four wild spp. namely *Musa acuminata* (A‐genome, 2*n* = 22), *M. balbisiana* (B‐genome, 2*n* = 22), *M. schizocarpa* (S‐genome, 2*n* = 22), and *M. troglodytarum* (T‐genome, 2*n* = 20) (Heslop‐Harrison and Schwarzacher, 2007). Wild bananas emerged from Southeast Asia, and over time, they diversified into various spp. and ssp. through geographical isolation across different continental regions and islands (Janssens et al., 2016a). Currently, banana cultivation spans across numerous countries, primarily in warm and humid tropical regions of the world that experience abundant rainfall (Table 1). These regions include Africa, Latin America, the Caribbean, Asia (south China, northern Vietnam, northeastern India, the Malayan Peninsula), and the Pacific (the Morobe province of Papua New Guinea and northern Borneo) (Nansamba et al., 2020; Mertens et al., 2021b).

**Table 1:** Geographical distribution patterns among diploid and triploid subgroups within cultivated bananas and plantains

Ploidy	Species	Group		Geographic distribution
Diploids	*M. acuminata*	AA		Philippines‐Melanesian, Papua New Guinea, Indonesia, and India
	*M. acuminata* × *M. balbisiana*	AB		South India
	*M. balbisiana*	BB		Myanmar, northeastern India, southwestern China
	*M. acuminata* × *M. schizocarpa*	AS		Papua New Guinea, Malayan Peninsula
	*M. troglodytarum*	AT		Papua New Guinea, Philippines, the island of Pohnpei
Triploids	*M. acuminata*	AAA	Cavendish	Great Lakes region in East Africa
			EAHBs	Great Lakes region in East Africa
			Mutika/Lujugira	Uganda
	*M. acuminata* × *M. balbisiana*	AAB	Plantains	West Africa, Humid tropical forest of America, and South India
			Silk	Caribbean and South East Asia
			Mysore	Brazil, a few restricted areas of Mexico and Venezuela, and the southern states of India
			Pome	Australia
		ABB	Bluggoe	South America, especially Indigenous people of the Amazon basin and the savannas
			Pisang Awak	Backyard garden of Asia
		AAB	Maia Maoli/Popoulu	West coast of South America, Pacific islands

Bananas have been selectively bred for desirable traits such as seedlessness and enriched pulp through parthenocarpy. As a result, cultivated varieties (vars.) are predominantly propagated vegetatively. While this mode of propagation ensures trait stability, it also limits genetic diversity within and among cultivars (cvs.). The resulting genetic uniformity makes cultivated bananas particularly vulnerable to pests and diseases, including the highly destructive *Fusarium* wilt (FW). Furthermore, research on bananas has long been constrained by challenges such as their long life cycle, sterility, and reliance on parthenocarpy (Mohandas and Ravishankar, 2016). These factors have significantly hindered breeding efforts and complicated the development of effective strategies to combat virulent pests and diseases. The first banana genome, representative of *M. acuminata*, was sequenced in 2012, thereby allowing a wide array of studies to investigate diverse research areas comprising of gene family analyses (Cenci et al., 2014; Hu et al., 2015; Miao et al., 2020; Backiyarani et al., 2022a), chromosome structural variations (SVs) (Martin et al., 2017), plant genome evolution (Garsmeur et al., 2014; Sass et al., 2016; Wendel et al., 2016), genetic diversity (Christelová et al., 2017), association genetics (Sardos et al., 2016; Nyine et al., 2017, 2019), trait‐phenotyping (Van et al., 2018), and genetic engineering (Naim et al., 2018). In addition, the accessibility of advanced tools and data resources has paved the way for new revelations in assimilating and shaping the banana genome, using multi‐omics (Backiyarani et al., 2022b), machine learning (ML) (Singh et al., 2022), and gene editing (GE) (Tripathi et al., 2022) in the field of banana genetics and breeding. The effect of these novel insights and resources for banana improvement, particularly genetic engineering of genes controlling desirable traits, has accelerated the pace of banana breeding.

In this review, we will render a summary of banana (*Musa*) genome sequences and explore the astounding breakthroughs in banana genomics facilitated by high‐throughput technologies. Moreover, a rigorous appraisal of banana domestication and contemporary strides in banana improvement achieved by genomics, genetic engineering, and functional genomics including the cutting‐edge innovations designed to cope with FW will also be discussed. Concisely, this review accentuates the milestones in banana genetics, genomics, and breeding, and also proposes the directives for the development of new superior banana cvs.

## ORIGIN AND DOMESTICATION

Through the ages, the course of domestication holds an eminent position in the long‐standing history of plant evolution (Doebley et al., 2006; Morris et al., 2018). Co‐evolutionary relationships delineate that plant domestication was instigated over the last 20,000 years due to a captivating interplay between human communities and the plants themselves (Meyer and Purugganan, 2013; Gaut et al., 2018; Huang et al., 2022; Sardos et al., 2022). Cultivated bananas depict one of the earliest occurrences of plant domestication on Earth. The genus *Musa* originated and diversified in northern Indo‐Burma during the late Eocene. Dispersing from northwest to southeast, it disseminated into Southeast Asia and Oceania during the Oligocene and Miocene (Janssens et al., 2016a; Bebber, 2022).

**Figure 1:**
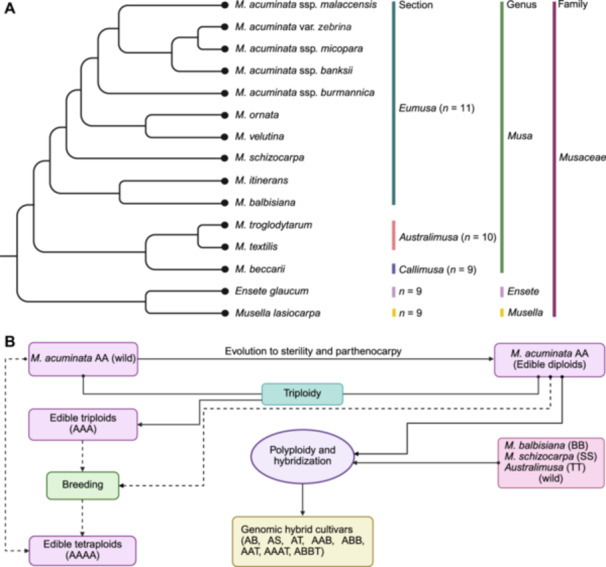
Phylogenetic relationships and evolutionary insights of *Musa* species and domesticated bananas **(A)** Phylogenetic tree illustrates the evolutionary relationships among *Musa* spp. and their wild relatives. **(B)** Evolution of domesticated bananas (adopted from Simmonds and Shepherd, 1955).

Based on morphological traits and basic chromosome number, the genus *Musa* was divided into five sections: *Eumusa* (*n* = 11), *Rhodochlamys* (*n* = 11), *Australimusa* (*n* = 10), *Callimusa* (*n* = 9), and *Ingentimusa* (*n* = 7). These five sections were widely accepted until 2013 when Häkkinen reappraised the five‐section system by integrating molecular phylogenetic evidence and proposed two infrageneric clade classification: section (sect.) *Eumusa* and sect. *Callimusa*. In this new system, sect. *Rhodochlamys* was synonymized with the sect. *Eumusa*, and sect. *Australimusa* and sect. *Ingentimusa* were treated as synonyms of sect. *Callimusa*; therefore, the two sections corresponded well to the basic chromosome number of *n* = 11 and *n* = 10/9/7, respectively (Li et al., 2010; Häkkinen, 2013) (Figure 1A). Later, in 2022, this classification was validated based on plastome sequence variations (Fu et al., 2022). One of the spp. from section *Eumusa*—*M. acuminata* (A)—is paramount in the cv. formation process and has been identified in all analyzed banana cvs. so far, with the exception of a small group of Fe'i cvs., which are exclusively derived from spp. within the *Australimusa* sect. (Simmonds, 1962; Li et al., 2013a; Martin et al., 2023). The domestication of bananas is believed to have originated through hybridizations involving *M. schizocarpa* and *M. acuminata* ssp. (specifically ssp. *banksii* and possibly ssp. *zebrina*) in New Guinea, more than 7,000 years ago (Martin et al., 2023). As early cvs. spread throughout Southeast Asia, additional hybridizations occurred with some other *Musa* spp. and ssp., including *M. acuminata* ssp. *zebrina*, *malaccensis*, *burmannica*, *halabanensis*, *M. balbisiana*, and at least one unidentified contributor, potentially a yet‐to‐be‐characterized *M. acuminata* ssp. (Martin et al., 2020, 2023; Sardos et al., 2022).

However, the accurate origin of triploid (AAA) bananas is still inconclusive. It is broadly acknowledged that these bananas are most presumptively the result of multiple triploidizations between different genotypes (Ploetz et al., 2007; Li et al., 2013a). Two leading triploid (AAA) bananas Cavendish and “Gros Michel” inherited their subgenomes from *M. acuminata* ssp. *malaccensis* (hereafter “Mam”), *banksii*, and *zebrina* (Li et al., 2013a). Correspondingly, it was presumed that the independent interspecific hybridization between *M. acuminata* and another wild spp.—*M. balbisiana—*located in northeast India and southeast China, had perhaps led to the emergence of unique diploid and triploid banana vars. (e.g., AB, AAB, ABB) (Simmonds and Shepherd, 1955; Baurens et al., 2019; Cenci et al., 2021). To illustrate, *M. acuminata* ssp*. banksii* contributed to the course of triploid plantains (AAB) domestication in the border zones between The Philippines and New Guinea, by passing on the maternal genome to the diploid cv. (AA), followed by the introduction of the B‐genome into present‐day triploid plantains (AAB) (Perrier et al., 2011; Li et al., 2013a). Later, these triploid plantains were migrated to the Pacific islands and Africa. In contrast, *M. balbisiana* donated the maternal genome to some other triploid cvs. (AAB and ABB), like “Pisang Raja Bulu” and “Namwa Khom” (Li et al., 2013a).

Based on the updated classification system established on advanced molecular genetic approaches, a third wild sp.—*M. schizocarpa*—from New Guinea, might have contributed to certain minor cvs. (AS). This contribution occurred as a result of multiple hybridizations of *M. schizocarpa* with AA cvs. (Carreel et al., 2002; Němečková et al., 2018). To exemplify, a modern cv. “Wompa” (AS) inherited the maternal genome from *M. schizocarpa*, while the paternal genome was donated by *M. acuminata* ssp. *banksii* through interspecific hybridization between these two spp. (Li et al., 2013a). Likewise, Fe'i bananas were found in New Guinea and Polynesia and some rare interspecific cvs. (AT) were derived from another wild sp.—*M. troglodytarum* L.—from *Australimusa* sect (De Langhe et al., 2009; Li et al., 2013a) (Figure 1B). It is clear that, unlike the A‐genome and the B‐genome, the S‐genome and T‐genome cvs. only exist in New Guinea (Li and Ge, 2017).

**Figure 2:**
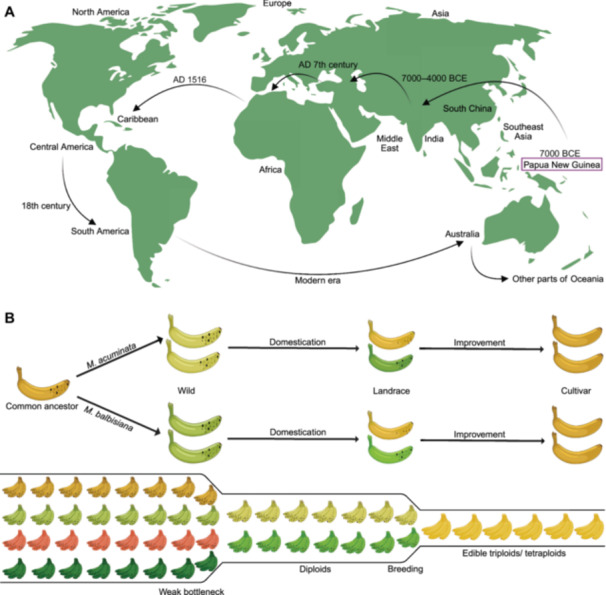
Origin and selection during domestication and improvement of bananas **(A)** Origins and global dispersal patterns of bananas across historical epochs: A holistic exploration (Mohandas and Ravishankar, 2016). **(B)** A schematic diagram rendering the phenotypic and genetic diversity during domestication and improvement of bananas. The domestication process of bananas has faced a weak bottleneck, leading to marginal diversity reduction, while the improvement process depicts a marked reduction in diversity.

Cultivated bananas, from their origin of domestication, eventually scattered toward the Middle East, Africa, and nearby areas, perhaps in the ad seventh century. Later, in AD 1516, bananas were acclimated in Central, Caribbean, and tropical regions of America. During the 18th century, bananas journeyed toward South America, the Pacific, and some parts of Oceania (Seymour, 1995) (Figure 2A). Following on, the contemporary studies on spp. distribution using biogeographic data advocate the plenitude of banana spp. in northeast India, northern Indo‐Burma, peninsular Malaysia, Sulawesi, lowland Borneo, Papua New Guinea, humid tropical forests of America, and Africa (Mertens et al., 2021b) (Table 1). Archaeology and the genetics of bananas have indicated that this global expansion of *Musa* spp. is an upshot of human migrations. Moreover, these migrations, together with the interplay between different *Musa* genome groups, have also assisted in the modeling of hybrid diploid and triploid genotypes (Perrier et al., 2011).

The dominant traits that banana plants selected during the course of domestication are parthenocarpy—fruit development without pollination—sterility followed by the production of seedless edible bananas, and multiple levels of ploidy (diploids, triploids, and tetraploids) (Simmonds, 1962; Denham et al., 2020). Hybridization had been instrumental in the election of these traits, with unstable meiosis producing diploid gametes at times that could generate triploid or tetraploid hybrids (Figure 2B). Furthermore, most edible bananas, either autotriploids (Cavendish‐AAA) or allotriploids (plantains‐AAB and cooking bananas‐ABB), contribute to their complex genetics. The release of *Musa* reference genome sequences has given research into banana evolution and domestication new insight (Davey et al., 2013; Martin et al., 2016). Consequently, sequencing‐based pioneering approaches have been instituted to meticulously find out the ancestral contributions in interspecific banana hybrids, delving into the ancestry mosaic patterns across the genome (Jeridi et al., 2011; Baurens et al., 2019; Martin et al., 2020; Cenci et al., 2021). These studies have demonstrated that the history of banana domestication is far more complicated than simply splitting the parental A‐genome and B‐genome. For instance, there are at least six ancestral groups participating in the Cavendish banana genome and every chromosome within the genome manifests a mosaic pattern, bearing sequences stemming from numerous sources (Martin et al., 2020). A recent study has explained the contribution of *M. schizocarpa* to many contemporary banana cvs., which was previously thought to be only embroiled in AS cvs. (Martin et al., 2023). An analogous paradigm has also been witnessed in the allopolyploids of other spp. (Gaeta and Chris Pires, 2010). Recent studies of a small set of banana cvs. with pure *M. acuminata* ancestries have revealed the presence of several ancestral gene pools that contributed to the development of banana cvs., which are absent in the current representation of wild *M. acuminata* diversity (Martin et al., 2020; Jeensae et al., 2021). However, the origins of these undefined gene pools, their contributions to the diversity of banana cvs., and their roles in the domestication and diversification of bananas remain unknown. The evolution under domestication of modern banana cvs. persists as a knowledge gap because the genomic structure and artificial selection of these cvs. remain considerably unexplored. Fortunately, advancements in third‐generation sequencing (TGS) techniques and the accessibility to genome sequences of different *Musa* spp. offer promising avenues to explore the origin and domestication of cultivated bananas on a broader genomic scale. Moreover, leveraging population genetics in the future will not only shed light on the origin of *Musa* but also offer valuable insight into the evolutionary history of many other significant crops. Simultaneously, if the complex origin of cultivated banana hybrids is ratified, it would require reconsidering current breeding strategies.

## CHALLENGES IN BANANA CULTIVATION: SPOTLIGHT ON *FUSARIUM* WILT

Together, parthenocarpy and sterility complicate banana breeding significantly, as creating high‐quality sterile and parthenocarpic vars. through the recombination of fertile and non‐parthenocarpic parents is quite difficult. Additionally, the presence of multiple ploidy levels among cvs. further restricts the available diploid parents for crossing, making the breeding process even more challenging. Furthermore, bananas are vulnerable to biotic and abiotic stress, leading to substantial economic and yield decline, which in turn influences food security. Major challenges to the cultivation of bananas and plantains arise from a range of pathogens, making bananas prone to several diseases induced by fungal (Sigatoka leaf spot, Black leaf streak, Eumusae leaf spot, and Panama disease (FW)), bacterial (Rhizome rot), and viral (Banana bunchy top virus (BBTV), Banana bract mosaic virus (BBMV), Banana streak virus (BSV)) infections, and insect pests and nematodes (Ploetz and Evans, 2015). The most disastrous disease in the history of bananas is FW, which ruined large banana plantations and reached pandemic status in the late 19th century (Ploetz, 2015; Dale et al., 2017a). *Fusarium* wilt is induced by a soil‐borne fungus belonging to the *Fusarium* spp., formerly categorized as *Fusarium oxysporum* f. sp. *cubense* (*Foc*) (Maryani et al., 2019). Subsequent to root infection, these pathogens colonize and block the plant's vascular system, resulting in acute wilting and ultimate plant death (De et al., 2000). *Foc* is typically categorized into three pathogenic races, that is, *Foc* race 1 (*Foc1*), *Foc* race 2 (*Foc2*), and *Foc* tropical race 4 (TR4), depending upon its compatibility with different groups of banana cvs. Meanwhile, *Foc* race 3 is considered non‐pathogenic, based on host susceptibility (García‐Bastidas, 2019). *Foc1* caused extensive damage to pre‐eminent cvs., that is, “Gros Michel,” “Pome,” “Silk,” and “Pisang Awak” in the 1950s (Ploetz, 2006). Conversely, *Foc2* exhibited compatibility with the cooking bananas subgroup “Bluggoe” (ABB). The third type, TR4, poses a severe threat to Cavendish plantations worldwide and various other banana vars. (García‐Bastidas, 2019). Lately discerned as a distinct sp.—*F. odoratissimum*—TR4 (Maryani et al., 2019) is pronounced to have originated from Southeast Asia (Chittarath et al., 2018; Hung et al., 2018; Zheng et al., 2018) and subsequently spread to various regions, including China (Li et al., 2013b), Mozambique (Butler, 2013), Jordan (García‐Bastidas et al., 2014), Lebanon (Ordoñez et al., 2016), Pakistan (Ordoñez et al., 2016), Turkey (Özarslandan and Akgül, 2020), and India (Damodaran et al., 2019), and to Africa and Columbia in South America (García‐Bastidas et al., 2020).

Over the years, multiple strategies have been trialed to combat this disease, such as disease‐free plantations, pertinent sanitation measures, biocontrol employment using *Trichoderma* spp. or non‐pathogenic *Foc*, and leveraging fungicides and fumigants (Chaves et al., 2016; Dita et al., 2018; Siamak and Zheng, 2018). Unfortunately, none of these approaches depicted complete efficacy in managing the disease, with the exception of the phenomenal resistance showcased by Cavendish clones against *Foc1* and *Foc2*. Steadily, the industry acceded these resistant clones that successfully coped with the *Foc1*‐ and *Foc2*‐induced epidemic, which had previously devastated “Gros Michel” bananas (Bubici et al., 2019). Likewise, diverse resistance levels to *Foc* are evident in multiple wild and cultivated bananas (García‐Bastidas, 2019), for example, Mam (“Pahang”), ssp. *burmannicoides* (“Calcutta 4”), and *M. itinerans* withstand TR4, while the cvs. “Rose” and “Tuu Gia” exemplify resistant edible bananas (Zhang et al., 2018; Zuo et al., 2018; García‐Bastidas, 2019). The broad cultivation of only one type of bananas –the Cavendish– raises concern due to its monoculture nature, in which it is primarily propagated through clones derived from suckers and tissue‐cultured plantlets. The incredibly finite genetic variations in such plantations make them extremely susceptible to potential disease epidemics. Furthermore, conventional breeding in this crop is also a weighty challenge. The genetic framework for resistance of Cavendish to TR4 remains obscure, regardless of its successful cultivation. Recently, a notable diminution in TR4 resistance genes has been ascertained within Cavendish compared with wild banana (Mam), offering potential objectives for molecular analysis of disease resistance in bananas (Huang et al., 2023).

However, these indispensable genetic resources are being untapped in modern banana breeding programs by leveraging genetic modification techniques, including protoplast electroporation (Sagi et al., 1994), particle bombardment (Sági et al., 1995), and *Agrobacterium*‐mediated transformation (May et al., 1995). Due to daunting obstacles to coping with TR4 through conventional breeding, researchers have commenced efforts to harness genetic engineering for controlling FW, leading to the development of copious *Foc*‐resistant transgenic banana vars. The perpetual advancements in genetic engineering and genomics techniques and the availability of banana genomes aim to assist further genetic innovations in bananas (D'hont et al., 2012; Maxmen, 2019; Wang et al., 2019), which will be further elaborated on in the upcoming sections. However, the continuous immune response triggered by genetic engineering leads to a decline in yield. To address this issue, there is a need to orchestrate functional genetic experiments on the *Musa* genomes that will reveal the molecular mechanisms behind disease resistance in bananas.

## MULTI‐OMICS APPROACHES FOR BANANA IMPROVEMENT

**Figure 3:**
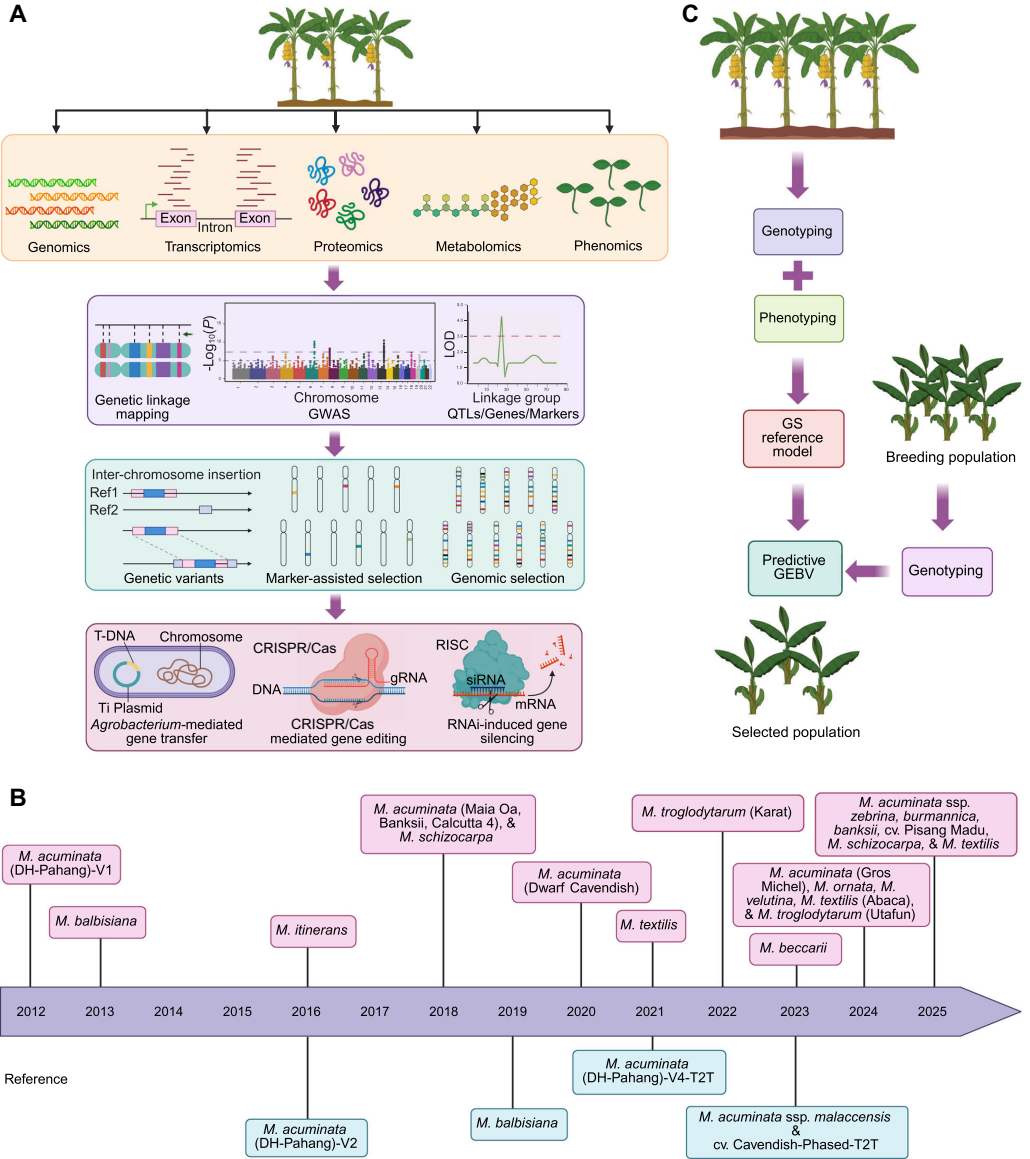
Multi‐omics approaches for banana improvement **(A)** Schematic diagram of multi‐omics approaches, genetic improvement platforms, and tools for bananas. **(B)** Headway of genome sequencing for different *Musa* spp. over the last two decades. **(C)** Schematic diagram of various stages of genomic selection for banana improvement. GEBV, genomic estimated breeding value; QTLs, quantitative trait loci; T2T, telomere to telomere.

Bananas—tropical fruits with a truncated shelf life—are ideal for fresh consumption and processing. However, traditional breeding methods, their uncertain genetic background, the restricted scope of focused genetic improvement, and problems like salinity, drought, and wilt diseases present substantial challenges to banana breeders and farmers. Moreover, designing disease‐resistant and high‐yielding banana vars. with uniform fruit shapes, appealing pulp, aroma, peel color, and high nutritional values is imperative to meet consumer demands. By deploying molecular breeding techniques and harnessing information on whole genome sequences, genetic linkage maps, molecular markers, transcriptome, and proteome and metabolome snapshots, it is now feasible to minimize conventional breeding impediments. These approaches are incredibly advantageous in comprehending the functional roles of genes, transcription factors (TF), and mechanisms associated with fruit traits and resistance against biotic and abiotic stress. This section delves into the use of integrated “multi‐omics” approaches to gain insight into important agronomic traits and their enhancement in bananas (Figure 3A).

The genome size of *Musa* varies between 468 megabases (Mb) and 619 Mb, as different wild spp. and cvs. exhibit diverse genomic sizes. Efforts to sequence *Musa* genomes were pioneered more than a decade ago and, in 2012, the first draft reference genome (A‐genome) representative of Mam (double haploid (DH) “Pahang”) was released (D'hont et al., 2012). This reference genome was a major breakthrough in banana genomics, genetics, and breeding. Since then, this genome has been revised twice (Martin et al., 2016; Belser et al., 2021). The draft version (V1) of “DH‐Pahang” genome was sequenced using 454, Sanger, and Illumina sequencing (D'hont et al., 2012) and in total 24,425 contigs and 7,513 scaffolds were assembled for this genome, rendering 90% of the predicted genome size (523 Mb) with 20.5× coverage. Hitherto, two upgraded versions (V2 and V4) of this draft genome have been released. In 2016, Illumina long‐insert sequences, a low contiguity optical map along with a denser genetic linkage map, were appended in V2 (Martin et al., 2016). The quality and completeness of V1 were improved by shrinking the overall count of scaffolds from 7,513 to 1,532, plus a substantial portion, accounting for 89.5% of the assembly (439 Mb), was anchored to the 11 chromosomes compared with the former 70%. The release of V2 proffered a platform to conduct comprehensive assessments of genetic diversity, functionality, and evolutionary aspects of banana genomes. The third version of the “DH‐Pahang” assembly was an internal assembly and was not made publicly available to the scientific community.

Recently, with the emergence of TGS (Pacific Biosciences (PacBio) and Oxford Nanopore Technology (ONT)) technologies, the construction of more contiguous long sequences and complete assembly of banana genomes has been instigated. In 2021, a telomere‐to‐telomere (T2T) gapless chromosome‐scale genome assembly (V4) of this DH genotype was generated by ONT (Belser et al., 2021), and five out of 11 chromosomes were completely reconstructed in a single contig form, spanning from one telomere to the other. The contiguity of this assembly, in contrast with V1 and V4, was impressively upgraded, from 28 kilobases (kb) and 43 kb to 32 Mb, respectively. In total, 124 contigs were anchored to 11 chromosomes, covering approximately 90% of the estimated genome size, whereas V1 and V2 were mostly incomplete (55% and 70%, respectively). This breakthrough, for the first time, accentuated the specifics of the intricated composition of regions such as centromeres and clusters of paralogous genes within the *Musa* genome.

In 2013, a draft genome sequence of *M. balbisiana* (B‐genome) was assembled and released, later upgraded as a chromosome‐scale assembly from a DH genotype in 2019, revealing that the B‐genome was ∼79% the size of the A‐genome (Davey et al., 2013; Wang et al., 2019). A 492.8 Mb long genome assembly was generated using PacBio long reads and Illumina paired‐end and mate‐pair reads with a contig N50 of 2 Mb. In addition, 87.27% (430 Mb) of the assembly and 94.0% of the genes were anchored to 11 chromosomes (Wang et al., 2019;). An ample sequence aberration between the B‐genome and A‐genome was ascertained, with the occurrence of one homozygous single nucleotide polymorphism (SNP) per 23.1 base pairs (bp) and a pronounced level of heterozygosity synchronized with one heterozygous SNP per 55.9 bp. Moreover, mapping RNA‐seq data from triploid AAA and AAB hybrids to both genomes supported the hypothesis that interspecific recombination between homologous A and B chromosomes had occurred in *Musa* hybrids throughout their evolutionary history.

In 2018, the first chromosome‐scale genome of *M. schizocarpa* (S) was assembled leveraging ONT, Illumina, and Bionano sequencing technologies. The 587 Mb final assembly had a contig N50 of 6.5 Mb, and 98.3% of the assembled genes (32,809) were anchored to all 11 chromosomes. Compared with the A‐genome and B‐genome, the S‐genome depicted a high degree of synteny with the A‐genome, exhibiting only minimal rearrangements (Belser et al., 2018). This assembly was subsequently improved in 2025, achieving an enhanced contig N50 of 20.7 Mb and annotating 35,075 protein‐coding genes (Martin et al., 2025).

In 2021, the draft genome of *M. textilis* cv. “Abaca” (T‐genome) was generated using Illumina and PacBio reads, with an assembled length of 587 Mb covering 95.28% of the estimated genome size (Galvez et al., 2021). In terms of assembly quality statistics, this genome has a low scaffold N50 (47,291) and a low proportion of complete BUSCOs (78.2%) (Galvez et al., 2021). A year afterward, the *M. troglodytarum* (another T‐genome) genome based on ONT and PacBio sequencings was released, with a size of 603 Mb anchored to 10 chromosomes comprising 37,577 annotated genes. This genome assembly has provided evidence of fusion of parental chromosomes 8 and 9, along with a number of translocations and inversions within the T‐genome, as a result of divergence from their most recently shared ancestors (Li et al., 2022a). Recently, chromosome‐level genome assemblies of two representative accessions of the T‐genome, “Abaca” (*M. textilis*) and “Utafun” (*M. troglodytarum*), have been reported (Zhou et al., 2024). The quality and accuracy of these two assemblies are much better than that of the previously reported T‐genomes. The two genomes harbor 10 chromosomes with a total size of 613 and 619 Mb, respectively, which are the largest among all known Musaceae assemblies. The larger size of T‐genomes, compared with the A‐, B‐, and S‐ genomes, is mainly attributable to rapid expansion and slow removal of transposons. The significantly expanded gene families in “Abaca” might explain the cell wall thickness and mechanical properties in “Abaca,” while those expanded in “Utafun” were associated with embryo development. In addition to these, draft assemblies of other Musaceae spp. including *M. itinerans* (Wu et al., 2016), *E. ventricosum* (Harrison et al., 2014), and *M. beccarii* (Wang et al., 2023), and some ssp. of *M. acuminata*, namely ssp. *banksii*, *zebrina*, and *burmannica*, cv. and “dwarf Cavendish” (Rouard et al., 2018; Busche et al., 2020) have also been published during the past few years. Recently, chromosome‐scale genomes of *M. ornata* and *M. velutina* (Xiao et al., 2024), and upgraded genomes of *M. acuminata* ssp. *zebrina*, *burmannica*, and *banksii*, and *M. textilis* have been released (Martin et al., 2025) (Figure 3B; Table 2).

**Table 2:** Comparative statistics of published wild and cultivated *Musa* genome assemblies

Species/Cultivar	Section	Chr. No.	Technology	Size (Mb)	Contig N50 (Mb)	Genes	BUSCO (%)	Version	References
*M. acuminata* ssp. *malaccensis*	*Eumusa*	11	ONT	468.82	32.00	36,769	98.80	V4	Belser et al., 2021
*M. acuminata* ssp. *malaccensis* *	*Eumusa*	22	PacBio, ONT, Hi‐C	940	43.10	79,158	98.57	V1	Liu et al., 2023
*M. acuminata* ssp. *zebrina*	*Eumusa*	11	ONT	547.80	7.20	44,702	81.70	V1	Li et al., 2024
*M. acuminata* ssp. *zebrina*	*Eumusa*	11	Illumina, ONT	535.90	8.30	34,451	98.70	V2	Martin et al., 2025
*M. acuminata* ssp. *banksii*	*Eumusa*	11	Illumina, ONT	468.60	1.50	35,669	98.00	V1	Martin et al., 2025
*M. acuminata* ssp. *burmannica*	*Eumusa*	11	Illumina, ONT	477.40	3.80	35,669	98.70	V1	Martin et al., 2025
*M. acuminata* cv. Pisang Madu*	*Eumusa*	22	PacBio	965.70	28.80	72,425	98.80	V1	Martin et al., 2025
*M. acuminata* cv. Baxijiao*	*Eumusa*	33	PacBio, ONT, Hi‐C	1,424	44.80	91,526	96.40	V1	Huang et al., 2023
*M. acuminata* cv. Gros Michel*	*Eumusa*	33	PacBio	1,331	1.30	120,653	96.90	V1	Li et al., 2024
*M. schizocarpa*	*Eumusa*	11	Illumina, ONT, Bionano	525.30	6.50	32,809	97.60	V1	Belser et al., 2018
*M. schizocarpa*	*Eumusa*	11	Illumina, ONT	509.80	20.70	35,075	98.70	V2	Martin et al., 2025
*M. ornata*	*Eumusa*	11	ONT	477.18	12.88	39,177	98.08	V1	Xiao et al., 2024
*M. velutina*	*Eumusa*	11	ONT	496.23	18.18	31,256	98.51	V2	Xiao et al., 2024
*M. balbisiana*	*Eumusa*	11	ONT	492.80	2.00	35,148	96.90	V2	Wang et al., 2019;
Plantain*	*Eumusa*	33	Illumina, PacBio, Hi‐C	1,690	2.01	88,078	92.00	V1	Xie et al., 2024
Silk*	*Eumusa*	33	Illumina, PacBio, Hi‐C	1,520	2.92	94,988	92.00	V1	Xie et al., 2024
*M. troglodytarum* cv. Karat	*Australimusa*	10	Illumina, PacBio	603.60	5.10	37,577	97.70	V1	Li et al., 2022a
*M. troglodytarum* cv. Utafun	*Australimusa*	10	Illumina, PacBio	619	3.60	36,080	94.30	V1	Zhou et al., 2024
*M. textilis* cv. Abaca	*Australimusa*	10	ONT	613	3.50	35,077	96.90	V1	Zhou et al., 2024
*M. textilis*	*Australimusa*	10	Illumina, ONT	531.10	4.00	33,662	95.80	V2	Martin et al., 2025
*M. beccarii*	*Callimusa*	9	ONT	569.60	67.10	39,112	98.40	V1	Wang et al., 2023

Abbreviations: BUSCO, Benchmarking Universal Single‐Copy Orthologs; ONT, Oxford Nanopore Technology; PacBio, Pacific Biosciences.

* This is a haplotype‐resolved genome assembly.

In order to develop a comprehensive genetic profile that can unravel the inherent complexity of a fruit crop, it is crucial to generate a haplotype‐phased reference genome (Shi et al., 2023; Su et al., 2024). However, there has been no high‐quality haplotype‐resolved reference genome due to the intricacies of banana genomes. Very recently, the first‐ever phased T2T reference genomes of diploid (AA) Mam and triploid (AAA) “Baxijiao” a Cavendish cv. of bananas have been successfully released (Huang et al., 2023; Liu et al., 2023). Moreover, phased assemblies of triploid (AAA) Cavendish and “Gros Michel” (Li et al., 2024), two allotriploid (AAB) cultivated bananas: Plantain and Silk (Xie et al., 2024), and a diploid hybrid cv. “Pisang Madu” (Martin et al., 2025) have also been reported (Table 2). These phased reference genomes will stand as invaluable genetic assets for further discernment of the subgenome evolution of *Musa* spp. and molecular breeding and genetic improvement of bananas. The genome assemblies, annotations, and the comprehensive 'omic resources of most of the bananas and banana relatives (high‐quality and draft sequences) are stored in the Banana Genome Hub (BGH; https://banana-genome-hub.southgreen.fr) (Droc et al., 2022).

A single or several reference genomes cannot utterly capture the entire genetic diversity within a genus. So, the emergence of inexpensive and readily accessible long‐read sequencing technologies and progressions in bioinformatics have expedited the transformation of single reference genomes for many crops into linear pangenomes and, more recently, graph‐based pangenome approaches (Zhang et al., 2021a; Li et al., 2022b; Liu et al., 2024). The notion of a pangenome entails all the genome sequences identified within a specific sp. under scrutiny. Early linear pangenomes implied the integration of multiple genome assemblies or the coalescence of the reference genome with additional assembled sequences from unaligned reads from various populations. Concomitantly, a graph‐based pangenome is proficient in incorporating intricate variants from multiple genomes of the target spp. into a single graphical illustration, making it adequate for future population genetic analyses (Khan et al., 2020). More recently, the first‐ever cross‐genus pangenome of bananas including 15 representatives of *Musa* and *Ensete* has been reported (Rijzaani et al., 2022). In addition to the 35,276 gene models previously known to exist in the Mam genome, 12,310 newly identified candidate protein‐coding gene models were discerned. When compared with previous studies on plant pangenomes, the banana pangenome manifested a significantly higher proportion of additional genetic sequences. One possible rationale for this phenomenon is the inclusion of multiple spp. from both genera into the banana pangenome, which brought about an expansion of genetic diversity and an increase in the pangenome's overall size. Moreover, the gene presence–absence variations (PAVs) emphatically separated *Musa* from *Ensete*, with further disparities observed within the *Musa* species. The genetic deviation between both genera contributed to the ascertainment of genes involved in evolution and selection, along with essential functions and traits like drought tolerance. Most of the *Ensete* spp. are known for their drought tolerance, therefore finding out the genomic basis of this significant trait could potentially bring about an improvement in the *Musa* spp. with regards to drought tolerance.

The accessibility of high‐quality *Musa* genomes has increased the wide range of studies exploring evolutionary history and breeding in bananas. Thanks to TGS, the gapless and T2T haplotype‐resolved chromosome‐scale assembly of diploid and triploid genotypes is in progress. These milestones offer unparalleled opportunities to unravel genomic regions previously inaccessible by short‐read technologies. These advancements can significantly influence future genetics and comparative genomics and contribute to improving banana crops worldwide.

*Genomic variation maps* are pivotal to understanding the evolution, adaptation, and improvement of plants. Advanced sequencing methods like NGS can unearth copious DNA sequence variants, such as SNPs, insertion–deletion mutations (InDels), and SVs. Genome‐wide variants in bananas have been studied to gain insight into the genetic diversity and relationships among different banana cvs. A digital catalog of high‐density markers for banana germplasm repertoires is available offering valuable data on genetic variants and their connection to the diversity stored in the gene bank. The available datasets vary in size, ranging from 245,285 to over 7 million SNPs, depending on the specific study (Rouard et al., 2022). To attain a deeper understanding into the genetic variations within EAHBs, in total 38 triploid accessions were characterized using genotyping based on Simple Sequence Repeat (SSR) or Microsatellite Markers. The comparative analysis in the internal transcribed spacer region revealed a firm rapport between EAHB clones and Mam and, unexpectedly, *M. schizocarpa* (Němečková et al., 2018). Therefore, the review of NGS data from nine interspecific banana cvs. (diploids and triploids) revealed large chromosomal variations. Recombination between the A‐genome and B‐genome was seen across the entire chromosome length but was also influenced by large SVs in specific regions. A comparison of the A‐genome and B‐genome chromosome structures showcased the occurrence of two prominent SVs: a paracentric inversion on chromosome 5 and a reciprocal translocation encompassing chromosomes 1 and 3 (Baurens et al., 2019). Another genome‐wide variation study involving 226 Musaceae accessions (wild and cultivated) to find out the patterns of inter(sub)specific hybridization confirmed that the geographical origins of globally cultivated bananas could be traced back to New Guinea (Martin et al., 2023).

However, there is still a lack of understanding regarding the finding of InDels in bananas. The availability of *Musa* reference genomes is a cornerstone for further studies on genome‐wide variations in bananas. *Musa* genomes—particularly Mam—hold approximately 52.6% (~246 Mb) of transposable elements (TEs) (Belser et al., 2021), and landraces and wild banana spp. both revealed hefty levels of heterozygosity, suggesting the presence of different gene versions at the same loci. These phenomena significantly contributed to the genetic diversity observed within the *Musa* species. Persistent endeavors to upgrade the genome assemblies and annotations are needed to improve our mastery of genetic diversity and variations in banana cvs.

*Transcriptomics*—the study of all the transcripts residing in a single cell or a group of cells during a particular developmental stage or physiological state—in contrast with other functional genomic studies like proteomics, is a comparatively simple approach. It represents one of the initial phases of the transition from static genetic information to dynamic functional processes, and the molecular structure remains precisely connected to that of the genome (Kissel and Carpentier, 2016). Transcriptome profiling is a robust approach that quantifies mRNA expression levels through NGS technology, enabling the recognition of significant genes involved in diverse biological pathways (Fan et al., 2018a). A pioneering advancement in transcriptome analysis for numerous plants involves using high‐throughput RNA sequencing (RNA‐seq) technology, which has significantly benefited the interpretation of functional genes (Chen et al., 2014). For bananas, RNA‐seq holds weighty prospects for upgrading genome annotations and precise ascertainment of gene loci, intron‐exon structures, and splice variants, and mapping of SNPs.

Transcriptomic data from different *Musa* spp. have been released (Wang et al., 2019; Busche et al., 2020; Sambles et al., 2020), providing insight into biological pathways underlying important agronomic traits. For instance, transcriptomic analysis was used to understand the role of *MusaPIP2;6*—a salt stress‐responsive gene—in bananas, propounding its association with improved photosynthetic efficiency and reduced membrane breakage (Sreedharan et al., 2015). Moreover, an RNA‐seq‐based comparative transcriptomic analysis between cold‐intolerant bananas and cold‐tolerant plantains was applied to investigate the cold‐tolerance pathway. It came to light that plantains exclusively turn on the cold‐tolerance pathway through the regulation of *ICE1* and *MYBS3* expression. This rapid activation and specific induction of *ICE1* and *MYBS3*, along with other cold‐related genes, are considered to be the major contributors to plantains' superior cold tolerance when compared with bananas (Yang et al., 2015). A similar kind of analysis focusing on TR4 resistance was carried out between the resistant wild relative *M. acuminata* ssp. *burmonicoides* and the susceptible cv. “Brazilian.” The results indicated that the high resistance observed in *burmonicoides* was primarily attributed to the overexpression of a handful of defense‐related genes (Li et al., 2020). Furthermore, the phenotypic and transcriptomic data from cv. “Brazilian” and its dwarf mutant were compared to discover plant height‐related genes. Within the gibberellin (GA) and indole acetic acid (IAA) signaling pathways, 13 and 7 candidate genes, respectively, were identified. These genes exhibited lower expression levels in the dwarf bananas, which aligned with the findings from the transcriptome analysis (Cai et al., 2023). In addition, a transcriptomic study unveiled the substantial involvement of TF MYB108 and WRKY75 in steering the growth and development of banana leaves and roots during prolonged magnesium deficiency (Yang et al., 2021). In the same area, several studies have been carried out to probe the response mechanisms for abiotic and biotic stress (Backiyarani et al., 2015; Zorrilla‐Fontanesi et al., 2016; Gamez et al., 2019; Xu et al., 2019; Wei et al., 2022; Htwe et al., 2023). The comprehensive findings of these studies have accentuated the power of high‐throughput molecular profiling techniques, offering great potential to advance our future understanding of other agronomic banana traits.

*Small RNAs* are non‐coding RNAs, for example, microRNAs (miRNAs), which are approximately 21–24 nucleotides long, and responsible for controlling the expression levels of specific target genes primarily through endonucleolytic cleavage or translational inhibition (Budak and Akpinar, 2015). These miRNAs are indispensable for the prolific physiological aspects of plants such as fruit ripening, development, growth, and response to abiotic and biotic stress. In bananas, miRNA169, miRNA156, and miRNA2188 were found to be upregulated in leaves during drought stress (Muthusamy et al., 2014). More recently, miRNA screening has been upgraded with advancement in NGS and high‐throughput sequencing approaches. For instance, among the 128 miRNAs found in bananas by leveraging high‐throughput sequencing, 22 have shown differential expression in response to ethylene. These miRNAs were found to tap into genes encoding proteins like GATA, ARF, DLC, AGO, and others (Dan et al., 2018). Another study reported that 46 miRNAs were upregulated, while 25 miRNAs were downregulated during banana fruit ripening. The primary target genes of these miRNAs were associated with miRNA biogenesis, fruit softening, and aroma biosynthesis (Lakhwani et al., 2020). Long non‐coding RNAs (lncRNAs) discovered in bananas also were also linked to miRNAs associated with plant and seed development along with responses to abiotic and biotic stress (Sampangi‐Ramaiah et al., 2019). Similarly, some fruit softening and ripening and stress response‐associated miRNAs and lncRNAs have also been identified in bananas (Table 3) (Sheeba et al., 2013; Bi et al., 2015; Chai et al., 2015; Ghag et al., 2015; Lee et al., 2015; Liu et al., 2018a; Kong et al., 2022; Putra et al., 2023). However, the determination of miRNAs regulating various functions in bananas remains inadequate. So, delving into the myriad roles and regulatory mechanisms of miRNAs in bananas is imperative to augment their genetic enhancement and stress tolerance.

**Table 3:** List of non‐coding RNAs identified in Bananas

miRNAs identified	Targets	Traits	References
miRNA166	25	Viral infection response	Sheeba et al., 2013
miRNAs169, 156, and 2188	‐	Drought stress tolerance	Muthusamy et al., 2014
miRNA156	173,161	Leaf stunted growth	Ghag et al., 2015
miRNA167c	244	Tissue specificity	Chai et al., 2015
miRNAs—59	120	Salt stress tolerance	Lee et al., 2015
miRNAs—82	815	Ripening	Bi et al., 2015
miRNAs—22	12	Ripening	Dan et al., 2018
lncRNAs—12,462	‐	Cold stress tolerance	Liu et al., 2018b
miRNAs—46	944	Fruit softening and aroma biosynthesis	Lakhwani et al., 2020
miRNA172	1,060	Cold stress tolerance	Liu et al., 2018a
miRNAs393, 160, and 167	‐	Ripening	Kong et al., 2022

Abbreviations: miRNAs, micro RNAs; lncRNAs, long non‐coding RNAs.

*Proteomics*—an invaluable tool for studying the structures, functions, and interactions of the entire proteome in a biological system—allows the detection of differentially expressed proteins throughout the development and growth of organisms (Jorŕn et al., 2007). The proteome‐based approaches equip the scientists with the ability to scrutinize various isoforms and post‐translational events, providing valuable insight beyond other molecular levels. Unlike transcripts, proteins directly influence biological processes, so magnifying our comprehension of biological processes, particularly at the protein level, will raise the quality and relevance of acquired biological information, thereby assisting advancements in plant development. The advent of advanced high‐throughput techniques, such as two‐dimensional gel electrophoresis (2DE), mass spectrometry (MS), isobaric Tags for Relative and Absolute Quantitation (iTRAQ), and Tandem Mass Tags (TMT) has raised proteomic research to a new level.

For bananas, an in‐depth proteomic analysis was carried out, and the data for the identification of expression levels of an articulated stress‐responsive protein family HSP70 during osmotic stress were assembled. A 2DE MS/MS approach was deployed to divulge and measure the diverse paralogs and allelic variations present in the HSP70 spots (Vanhove et al., 2015). Furthermore, a multivariate examination of ripening‐related proteins was carried out to correlate them with starch dynamics, investigating fruit ripening in plantains and Cavendish bananas. The ripening process accounted for a diminution in sucrose synthase levels and an expansion in acid invertase levels, stipulating a shift in sucrose consumption. In addition, clear cv‐specific variations were observed in granule‐bound starch synthase, alpha‐amylase and beta‐amylase, and cell wall invertase when evaluating protein content at an equivalent stage of ripening. Additionally, dissimilarities in small heat shock proteins and the cell wall‐modifying enzyme xyloglucan endotransglucosylase/hydrolase endorsed the speculation of raised carotenoid content and firmer fruit texture, respectively, in plantains (Bhuiyan et al., 2020). Similarly, a proteome analysis for banana fruit softening leveraging iTRAQ technology was performed, and 420 differentially expressed secreted proteins, typically involved in cell wall metabolism, stress response and defense, signaling, and protein metabolism and modification, were discovered (Xiao et al., 2019). A comparison of the characterization of the fruit proteomes of dessert bananas and plantains separated the proteins that were merely encoded by the plantain genome. In total, 37 alleles covered by 59 peptides were found to be unique for plantains (Campos et al., 2018). In a recent study, the impact of drought stress on bananas and their subsequent recovery was investigated in cv. “Berangan.” Proteomic analysis revealed significant alterations in 274 proteins associated with energy, carbohydrate, and amino acid genetic information refinement, metabolism, and secondary metabolite biosynthesis (Chen et al., 2021). Moreover, molecular mechanisms related to osmotic and drought stress tolerance (van Wesemael, 2019; Amnan et al., 2021) and embryogenesis (Kumaravel et al., 2017) have also been investigated. Most of these studies reported the indispensable role of advanced proteomics technologies in deciphering novel proteins associated with plant responses and adaptations to different stress conditions. These findings produce ways to explore other banana traits at the protein level, providing valuable insight that could assist breeders in developing improved strategies to boost banana productivity.

*Metabolomics* involves an intricate analysis of endogenous metabolites, aiming to systematically discern and quantify these metabolites in response to different conditions and treatments within a single cell, tissue, or organ (Zhang et al., 2012). The chemical composition of bananas helps to determine their sensory properties and nutritional value. In banana fruits, small metabolites play pre‐eminent roles, particularly in fundamental metabolic processes. However, hitherto, despite their significance, the number of metabolomics studies carried out on bananas remains lacking. Metabolomics analyses of bananas during post‐harvest senescence using proton high‐resolution nuclear magnetic resonance, revealed the available primary and secondary metabolites, encompassing organic acids, amino acids, carbohydrates, and phenolics. Although bananas depicted identical chemical profiles at five senescence stages, there were still substantial, albeit minor, variations in the levels of individual compounds. The primary metabolites associated with post‐harvest senescence in bananas included valine, alanine, aspartic acid, choline, acetate, glucose, malic acid, gallic acid, and dopamine (Yuan et al., 2017). Another analogous study stressed that the high rates of glycolysis were likely to be prerequisites to support high carotenoid accumulation in banana pulp (Heng et al., 2019). A gas chromatography‐mass spectrometry‐based metabolomics approach was used to explore the impact of low temperature and chitosan treatment on bananas. The two treatments resulted in a delayed fruit ripening by postponing metabolite changes during the ripening process. Low temperature influenced putrescine, β‐alanine, and malic acid levels, possibly indicating hypoxia or the regulation of plant senescence through polyamines. Conversely, chitosan regulated acetyl‐CoA carboxylase, glycine, and α‐ketoglutaric acid, influencing hormone‐related processes and antioxidant activities (Parijadi et al., 2022). Additionally, the effect of λ‐carrageenan on plant growth was appraised, indicating that λ‐carrageenan promotes an increase in photosynthesis rate, the biosynthesis of protein and secondary metabolites, ultimately leading to growth stimulation (Thye et al., 2022). Moreover, the indispensable pathways contributing to uniconazole‐induced dwarfism in bananas were investigated by overlapping Kyoto Encyclopedia of Genes and Genomes annotations for differentially expressed genes (DEGs) and differentially abundant metabolites. This metabolome analysis unearthed significant variations in flavonoids in bananas treated with uniconazole. The increase in flavonoid and lignin biosynthesis might contribute to the dwarfing phenotype observed in banana plants (Qin et al., 2022). Regardless, these studies provide valuable insight into the mechanisms behind certain traits, yet further research is needed to broaden our understanding of banana metabolomics comprehensively.

## GENOMICS‐ASSISTED IMPROVEMENT OF BANANA AGRONOMIC TRAITS

*Genome mapping* institutes the directions of a genome, aiding in the identification and manipulation of essential genes, as well as discerning the molecular context of both coding and non‐coding DNA sequences (Jones et al., 1997). Before the assembly of the first banana genome, the main focus of banana research was on building frameworks and laying the foundation for genome mapping. The sterility of the important cvs. structural heterozygosity, polyploidy, the extended growth cycle of the crop, and the scarce supply of molecular tools had impeded the advancement of generating high‐density genome maps for *Musa* spp. (Sampangi‐Ramaiah and Ravishankar, 2016). The first linkage map for bananas was formulated in 1993 based on a second filial generation (F2) population of two diploid cvs. of *M. acuminata* (SF265 × banksii) revealing variation for parthenocarpy. The map constituted 90 loci (58 Restriction Fragment Length Polymorphisms (RFLPs), 28 Random Amplified Polymorphic DNAs (RAPDs), and four isoenzymes), covering 606 centiMorgan (cM). Out of these 90 loci, 77 were successfully placed into 15 LGs, while 13 segregated independently (Fauré et al., 1993). With the advent of NGS technologies, recent endeavors have witnessed a paradigm shift toward modeling high‐density genetic maps of *Musa* for various genetic backgrounds leveraging first filial generation (F1) and backcross populations (Table 4) (Noyer et al., 1997; Vilarinhos, 2004; Kayat et al., 2009). The number of molecular markers deployed in these genetic maps has increased from 100–200 to up to 1,000. Most of these studies accentuated the occurrence of chromosomal structural rearrangements, such as inversions and translocations, frequently seen within the genus *Musa*. In the year 2010, the first saturated linkage map—referred to as the reference *Musa* map—was released, deploying 180 individuals from the F1 population of two genetically distinct cvs. of *M. acuminata* (Borneo × Pisang Lilin). This map comprised 489 markers (167 SSRs (Simple Sequence Repeats) and 322 DArTs (Diversity Arrays Technology)), anchoring 11 linkage groups (LGs) and covering a total of 1,197 cM (Hippolyte et al., 2010). In addition, another population map comprising two maternal maps and a combined paternal map was formulated for *M. acuminata* segregating for *Radopholus similis* resistance. This map contained 231, 152, and 361 markers, respectively (including DArTs, SSRs, and allele‐specific polymerase chain reaction), anchored to 15 LGs, spanning 670 cM (Mbanjo et al., 2012). Recently, a genotyping by sequencing (GBS)‐based linkage map for an entirely novel mapping population was developed using progenitor lines with distinct genomic compositions (A‐genome and B‐genome). *M. acuminata* ssp. *burmannicoides* Colla (“Calcutta‐4”) and *M. balbisiana* (Bee hee kela) segregating for drought tolerance were crossed to produce an F1 mapping population of 96 individuals. The map comprised 1,015 SNP markers placed on 11 LGs. Its total length was 4,828.88 cM, with an average distance of 4.9 cM between markers (Sampangi‐Ramaiah et al., 2023). More recently, the evolution of TGS technologies has revolutionized the recognition of high‐quality molecular markers like SNPs in large populations, including natural populations. Consequently, the quality and resolution of genetic linkage maps have been considerably upgraded (Wu and Qin, 2019). A linkage map based on selfing of wild bananas (Mam) was drafted to look at the inheritance of resistance toward FW. GBS discovered 2,802 high‐quality SNPs, which were placed into 11 LGs. Interestingly, quantitative trait loci (QTLs) indicating partial resistance to TR4 were also identified at the same position (Ahmad et al., 2020).

**Table 4:** Molecular marker‐based linkage maps for bananas employing segregating mapping populations

Population	SF265 × *Banksii*	Selfed M53	Calcutta 4 × Madang	Selfed Mam	Borneo × Pisang Lilin	TmB2x 6142‐1 × TmB2x 8075‐7	Selfed Wild Mam	*M. acuminata* (Calcutta 4) × *M. balbisiana*
Population size	92	89	‐		180	81	225	96
Marker type	RFLP, RAPD, and Isozyme	RFLPs, SSRs, and AFLPs	RFLPs, AFLPs, and SSRs	AFLPs, RAPDs, and STMSs	SSRs and DArTs	DArTs, SSRs, and AS‐PCRs	SNPs	SNPs
F_1_/F_2_	F_2_	F_1_	F_2_	F_1_	F_1_	F_1_	F_2_	F_1_
Number of markers	90	> 300	120	367	489	744	2,802	1,015
LGs	15	18	14	69	11	31	11	11
Genome coverage (cM)	606	1,200	597		1,197	1,004	‐	4,828.88
Trait	Parthenocarpy	Black Sigatoka resistance	Translocations	*Foc* resistance	Translocations	Resistance to *Radopholus similis*	*Foc* resistance	Drought tolerance
References	Fauré et al., 1993	Noyer et al., 1997	Vilarinhos, 2004	Kayat et al., 2009	Hippolyte et al., 2010	Mbanjo et al., 2012	Ahmad et al., 2020	Sampangi‐Ramaiah et al., 2023

Abbreviations: AFLPs, Amplified Fragment Length Polymorphisms; AS‐PCRs, Amplification of Specific Alleles by Polymerase Chain Reaction; cM, centiMorgan; DArTs, Diversity Arrays Technology; *Foc*, *Fusarium oxysporum* f. sp. *Cubense*; F1, Filial 1; F2, Filial 2; LGs, Linkage groups; RFLP, Restriction Fragment Length Polymorphisms; SNPs, Single Nucleotide Polymorphisms; SSRs, Simple Sequence Repeats; STMSs, Sequence‐Tagged Microsatellite Sites.

*Quantitative trait loci* and the underlying genes governing agronomic traits must be meticulously explained for marker‐assisted breeding abetted by multi‐omics techniques. Genetic linkage maps are invaluable tools in the QTL mapping process for various fruit crops. Notably, QTLs associated with several banana traits have been successfully located on specific chromosomes through linkage maps. QTLs pertaining to TR4 resistance were found at the distal part of chromosome 10 at 0 and 4.35 Mb (Ahmad et al., 2020). A recent study discovered 12 significant QTLs associated with organoleptic quality traits, namely pulp acidity, firmness, and dry matter content, during banana ripening. The ones with the most substantial impact on pulp acidity were located on LGs 1–7 on the genetic map (Biabiany et al., 2022). However, in the year 2023, a SNP‐based QTL mapping unveiled five primary QTLs for drought tolerance (wax13_1, wax15_1, wax15_2, lwrc_1, and asd_1), located on chromosomes 2, 5, and 8. The percentage of phenotypic variance explained (PVE) of the identified QTLs ranged from 15.9% to 19.8% (Sampangi‐Ramaiah et al., 2023). Although banana QTLs may not be as numerous as those in some other crops, recent genomic advancements have paved the way to detect more QTLs for banana traits. Also, the QTLs identified through genetic linkage maps are often quite large, several megabytes, and contain numerous genes. Therefore, integrating supplemental approaches like genome‐wide association studies (GWAS), and discernment of DEGs and expression QTLs (eQTLs, also known as eGWAS), is paramount to more effectively pinpointing the target genes responsible for important agronomic traits in bananas.

*Genome‐wide association studies* speed up the identification of genotype–phenotype associations by evaluating differences in allelic frequencies of genetic variants among individuals of similar genetic ancestry who display contrasting phenotypes (Alseekh et al., 2021). This approach has been instrumental in uncovering numerous robust associations linked to a wide range of complex traits and diseases. Beyond association mapping, GWAS enables the explanation of the genetic architecture underlying phenotypic variation, including the estimation of heritability and the review of genetic correlations (Alseekh et al., 2021). Importantly, GWAS offers a comprehensive and unbiased genomic analysis framework capable of detecting common alleles with moderate effects on phenotypes (Hirschhorn and Daly, 2005). GWAS has been widely applied in many crops to identify candidate genes and QTLs using high‐density, genome‐wide markers (Tibbs Cortes et al., 2021). Developing high‐yielding banana vars. is essential to meet the food demands of a growing global population. Conducting GWAS in banana presents unique challenges due to changes from classical Mendelian genetic assumptions. First, most cultivated banana vars. are polyploid, which complicates association analyses due to the limited availability of suitable statistical models, and/or are interspecific hybrids, which introduces complexities in SNP mapping and increases genetic heterogeneity. Second, the variable fertility among banana cvs. significantly reduces the frequency of sexual reproduction, thereby limiting the available population size for association studies. Third, the vegetative propagation and high degree of clonal diversification in bananas can introduce confounding effects and lead to spurious associations arising from population structure. Despite these challenges, these features make the banana a compelling and complex model for genomic studies (Sardos et al., 2016).

In the last decade, GWAS has emerged as a valuable tool in banana research, despite persistent challenges related to polyploidy, clonal propagation, and interspecific hybridization. One early study analyzed 105 banana accessions using 5,544 SNP markers and identified 13 genomic regions associated with seedlessness through a mixed linear model (MLM), highlighting the feasibility of GWAS in this complex crop (Sardos et al., 2016). Efforts to dissect yield‐related traits have led to the identification of 25 loci associated with bunch weight components, primarily located on chromosome 3, based on 307 genotypes and 27,178 SNPs (Nyine et al., 2019). A more comprehensive investigation involving 124 phenotypically diverse accessions evaluated 12 traits related to morphology, fruit quality, and yield. This analysis yielded 187,133 SNPs mapped to the *M. acuminata* genome and 220,451 to *M. balbisiana*. By applying multiple association models, the researchers detected four significant marker–trait associations (MTAs) using MLM, and an additional 82 and 70 MTAs using Bayesian information criterion and linkage disequilibrium‐based models, respectively. Furthermore, they identified 38 and 40 candidate genes associated with the A‐genome and B‐genome, respectively (Rio et al., 2025). In another large‐scale study, a breeding population of 2,723 triploid hybrids, resulting from crosses between diploid and tetraploid *M. acuminata* parents, was evaluated across three crop cycles for 24 traits related to yield and plant, bunch, and fruit architecture. From a genotyped subset of 1,129 individuals, 205,612 SNPs were identified, and GWAS using MLM revealed one to five significant QTLs for 23 of the 24 traits, with many beneficial alleles originating from key ancestral lineages of modern banana cvs. (Hirschhorn and Daly, 2005). As banana genomics and breeding has advanced, the integration of refined GWAS approaches with cutting‐edge tools, like high‐throughput phenotyping, genome editing, and ML, offers immense potential to unlock the crop's complex genetic architecture and accelerate the development of superior cvs. to meet rising global demand.

*Marker‐assisted selection* (*MAS*) is considered a practical method for molecular marker‐based mapping of genes and accelerating the genetic improvement programs in fruit crops as many agronomic traits in these crops are complex and governed by QTLs (Collard and Mackill, 2008). However, despite the construction of several linkage maps for *Musa* spp., their quality and marker saturation are still limited, which impedes their full potential in banana breeding (Fauré et al., 1993; Noyer et al., 1997; Vilarinhos, 2004; Hippolyte et al., 2010; Mbanjo et al., 2012; Ahmad et al., 2020; Sampangi‐Ramaiah et al., 2023). In *Musa*, a handful of markers have been found to be associated with important agronomical traits, particularly disease resistance. For instance, methylation associated with resistance against black sigatoka disease was evaluated by identifying four methylation‐sensitive amplified polymorphism markers linked to the resistance gene (R gene) in cv. “Williams” (Gimenez et al., 2006). Similarly, a few RAPD markers have been used to elect banana clones that are salt‐tolerant and resistant to black sigatoka disease and nematodes (Miri et al., 2009).

Moreover, an *SERK*‐related SCAR marker associated with both somatic embryogenic competence and disease‐resistant response has also been identified (Huang et al., 2010; Cunha et al., 2015). Several FW related molecular markers, including RAPD (Javed et al., 2004; Kishor et al., 2018), SCAR (Wang et al., 2012, 2018; Cunha et al., 2015), and SSR (Rebouças et al., 2018) markers, have also been discerned. The availability of these molecular markers has contributed to accelerating the breeding progress in bananas. For example, hybridization approaches combined with molecular markers have vouched for the differentiation of infectious and non‐infectious alleles of endogenous BSV in *M. balbisiana*, resulting in the successful breeding of BSV‐free progeny. This breakthrough endows the development of enhanced banana hybrids while removing the potential risk of triggering infectious BSVs. This contributes to the creation of disease‐resistant banana vars. and promotes sustainable banana production (Umber et al., 2016).

*Genomic selection* (*GS*)—which falls under MAS—designs prediction models based on genome‐wide markers and phenotypic data from a training population (TP). These models are then used to forecast genomic estimated breeding values (GEBV) of a breeding population using solely genotypic information. The implementation of GS enables the selection of superior offspring at an early developmental stage, before phenotyping (Meuwissen et al., 2001). This approach escalates the breeding efficiency by truncating the breeding cycle and minimizing the time and cost associated with extensive phenotyping processes (Heffner et al., 2010). The GS approach is the most appropriate for choosing individuals with polygenic traits as it surpasses the limitations of MAS, which mainly concentrates on major QTLs, but is incapable of capturing the effects of minor genes (Desta and Ortiz, 2014). However, GS is still in its early stages when it comes to bananas (Figure 3C). To investigate trait variability and genetic diversity in bananas, a GS population comprising 307 genotypes with diverse ploidy levels was established. Genetic diversity within this TP was assessed using SSR markers. Additionally, the study aimed to determine the extent to which trait variation in bananas is influenced by factors such as cross combination, cropping cycle, field management practices, and their interactions with genotype (Nyine et al., 2017). Additionally, a TP consisting of 307 accessions with different ploidy levels and 10,807 SNPs was established for genomic prediction. The accuracy of the predictions for all 15 traits ranged from 76% to 84%, while the GEBV spanned from 0.04 to 0.76 (Nyine et al., 2018). Similarly, under drought stress conditions, GS demonstrated strong predictive accuracy for AAB banana fruit weight at maturity (3.41 ± 1.99 kg) and plant height (198.46 ± 12.66 cm) (Mbo Nkoulou et al., 2023). GS holds significant promise for banana development; however, further research is needed to improve the prediction accuracy before implementing GS.

The integration of GWAS and GS renders the substantial ability to accelerate genetic improvement in banana, particularly in complex polyploid cvs. such as Cavendish‐type bananas. To augment the efficiency of GS, GWAS has been incorporated into selection pipelines (GWAS–GS) by prioritizing SNPs with strong statistical associations to target traits. The inclusion of both validated and newly identified markers increases the biological relevance of the model and can significantly improve prediction accuracy (Chen et al., 2023a). This combined approach represents a promising strategy for advancing banana breeding efforts. GWAS facilitates the identification of major‐effect QTLs and key functional variants associated with critical traits such as disease resistance, yield, and fruit quality. Incorporating these trait‐associated loci into GS models can improve prediction accuracy and biological relevance by prioritizing markers with known effects. While GWAS focuses on detecting large‐effect variants, GS captures the cumulative influence of genome‐wide markers, including small‐effect loci, enabling effective prediction of breeding values for polygenic traits. This synergistic approach provides a powerful and efficient framework for accelerating banana breeding, helping to overcome challenges posed by the crop's clonal propagation and lengthy breeding cycles.

*Functional genomics*, which systematically investigates gene functions and their interactions within biological networks, has provided transformative insight into the genetic architecture of banana. Recent advancements in genomic technologies have enabled exponential growth in identifying and characterizing genes controlling important agronomic traits, along with discovery of their molecular regulatory mechanisms. Here, we summarize current knowledge through a comprehensive compilation of functionally validated *Musa* genes and their associated functions in Table 5, emphasizing newly discovered gene regulatory networks that are advancing banana genomics. The compiled data encompass genetic determinants governing parthenocarpy, fruit development, ripening, quality attributes, and both biotic and abiotic stress responses.

**Table 5:** Representative *Musa* genes with functionally validated roles in important agronomic traits

Trait category	Genes/Transcription factors (TF)	Functional annotation	References
Parthenocarpy	*MaAGL8*, *MaMADS16*, *MaMADS29*, *MaGH3.8*, *MaRGA1*, *MaEXPA1*, *MaGID1C*, *MaBAM1*, *MaHK2*	Regulation of natural parthenocarpy	Zheng et al., 2020
	*MaACLB‐2*, *MaZEP*	Regulation of both artificially induced and natural parthenocarpy	Zheng et al., 2020
Fruit development	MuMADS1, MaMADS7	TFs involved in flower and ovary development	Liu et al., 2009
	MaGST	TF active in various fruit development stages	Vaish et al., 2022
	*MaSWEET4b*, *MaSusy2.2*, *MaAPS1*	Total starch synthesis in fruit	Miao et al., 2017a; Miao et al., 2017b; Zhao et al., 2023
	*MaGBSSI‐3*	Amylose and resistant starch content regulation in fruit during development	Miao et al., 2014
	*MaSSIII‐1*	Starch granule structure and amylopectin accumulation	Miao et al., 2017c
	*MaSBE2.3*	Reduction in total starch and amylopectin content in fruit	Miao et al., 2020
Fruit ripening	*MA‐ACS1*, *MA‐ACO1*	Ethylene biosynthesis	Liu et al., 1999
	*MaIAA17‐like*	Chlorophyll degradation, starch breakdown, and cell wall modification	Chen et al., 2023
	*MbAMY‐2*, *MbAMY‐3*, *MbAMY‐8*, *MbBMY‐6*, *MbBMY‐8*, *MbDPE‐2*	Acceleration of the fruit ripening process by enhancing starch degradation	Wang et al., 2019
	MaMADS36	TF involved in the regulation of fruit ripening by controlling key carbohydrate and cell wall metabolism genes	Liu et al., 2018
	MaMYB, MaBEL1, MaCCCH33‐Like2	TFs regulate fruit maturation by activating genes related to cell wall and starch hydrolysis	Fan et al., 2018a; Song et al., 2023; Song et al., 2024
	*MaACS7/MbACS7*	Ethylene production during ripening	Wang et al., 2019
	MaERF012	TF controlling chlorophyll degradation, starch metabolism, and cell wall breakdown	Chen et al., 2022
	*MaERF9*	Ethylene response regulation in peel and pulp	Xiao et al., 2013
	*MaERF11*	Ethylene response regulation in peel and pulp	Xiao et al., 2013
Fruit quality	*MaGWD1*, *MaLSF2*, *MaBAM1*, *MaBAM2*, *MaBAM3c (MaBMY6)*, *MaBAM9b (MaBMY7)*, *MaBAM8*, *MaBAM10*, *MaDPE*, *MaAMY3*, *MaAMY3C*, *MaISA2*, *MaISA3*, *MapGlcT2‐2*, *MaSPS*, *MaSS*, *MaNI*, *MaAI*	Fruit starch degradation and sugar accumulation	Jourda et al., 2016; Miao et al., 2016; Fan et al., 2018a; Wang et al., 2019; Liu et al., 2022
	MabHLH6, MADS‐box, MaMYB16L, MaBEL1	TFs involved in starch degradation regulation	Xiao et al., 2018; Jiang et al., 2021; Liu et al., 2021; Song et al., 2023
	*MabHLH28*	Regulation of fruit softening	Wu et al., 2024
	*MaDXR1*, *MaPDS1*, *MaZDS1*, *MaSPL16*	Carotenoid biosynthesis at high temperatures	Zhu et al., 2023
	MaNAC029	TF involved in aroma compound synthesis	Wei et al., 2023
	MabZIP4/5	TF involved in aroma biosynthetic pathway activation	Guo et al., 2018
Biotic and abiotic stress	*2OGD*, *MaLYK1*, *LRR‐RLP*, *MYB36*, *PR*, *Macma4_11_g19760*	Disease resistance mechanisms	Zhang et al., 2019a; Álvarez‐López et al., 2022; Chelliah et al., 2023; Li et al., 2023b; Tian et al., 2023; Anuradha et al., 2024
	*MaWRKY24*	*Fusarium* wilt Tropical race 4 susceptibility	Liu et al., 2019
	*MaAQP*, *MaAGPase*, *MaMPK5*, *MAPKKK*, *MAPKK*, *MaCCO*, *MaU‐box*, *LysM*, *SRO*, *LOX*, *Expansin*, *MKK2* family genes, *MaROP5g*, *MaCCS*, *MaGHMP*, *WOX*, *RGA2*	Abiotic stress response activation	Dale et al., 2017a; Feng et al., 2016; Hu et al., 2017; Miao et al., 2018; Zhang et al., 2019a; Tak et al., 2021; Backiyarani et al., 2022a; Chaudhary et al., 2022; Chaturvedi et al., 2023; Fan et al., 2023; Wang et al., 2023a; Wang et al., 2023b; Zeng et al., 2023; Anuradha et al., 2024
	*MaPSY*, *MusaDHN‐1*, *MusaWRKY71*, *MusaNAC042*, *MusaNAC29*, *MaPIP2‐7*, *MaDREB1F*	Abiotic stress tolerance enhancement	Shekhawat et al., 2012; Kaur et al., 2017; Jangale et al., 2019; Tak et al., 2021; Chaudhari et al., 2023; Negi et al., 2023; Xu et al., 2023

One of the central focuses in banana research has been the genetic control of parthenocarpy, a trait of considerable agronomic importance due to its role in seedless fruit production. Potential target genes for investigating natural parthenocarpy in banana have been identified, including *MaAGL8* and *MaMADS16*, which regulate natural fruit development, and *MaACLB‐2* is expected to play roles in both artificially induced and natural parthenocarpy (Zheng et al., 2020). The molecular regulation of banana fruit development has received extensive attention owing to its key role in determining fruit set, size, structure, and quality. Flower and ovary development, which are tightly tethered to parthenocarpic fruit formation, are regulated by MADS‐box TFs, such as MuMADS1 and MaMADS7, which are dominantly expressed during these critical developmental stages (Liu et al., 2009, 2015). To date, many vital genes and TFs have been studied extensively for their roles in orchestrating the synthesis, breakdown, and remodeling of carbohydrates during banana fruit development and ripening. For example, *MaSWEET4b*, *MaSusy2.2*, and *MaAPS1* are associated with starch synthesis in banana fruit (Miao et al., 2017a, 2017b; Zhao et al., 2023). High expression levels of *MaGBSSI‐3* have been consistently correlated with amylose and resistant starch accumulation (Miao et al., 2014). In addition, the overexpression of *MaSSIII‐1* results in altered starch granule morphology and increased amylopectin content (Miao et al., 2017c), while silencing *MaSBE2.3* leads to significant reductions in total starch and amylopectin content in banana fruit (Miao et al., 2020).

Fruit ripening is a vital trait in banana, as it profoundly affects edibility, market value, shelf life, and overall consumer appeal. Fruit ripening is precisely regulated by a suite of TFs and metabolic genes that coordinate the biochemical and structural changes occurring during ripening. For instance, MaERF012 functions as a transcriptional activator that governs the expression of genes related to chlorophyll degradation, starch metabolism, and cell wall disassembly (Chen et al., 2022). Moreover, during ripening or in response to ethylene treatment, the *MaERF9* gene is upregulated, whereas the *MaERF11* gene is downregulated, specifically in the peel and pulp tissues (Xiao et al., 2013). Another gene, *MaIAA17‐like*, influences ripening by regulating genes involved in chlorophyll breakdown, starch catabolism, and cell wall modification (Chen et al., 2023b). Furthermore, the TF MaMADS36, with an ovate family protein MaOFP1, regulates fruit ripening by modulating the expression of genes involved in carbohydrate metabolism and cell wall modification (Liu et al., 2018). Additional regulators, such as MaMYB, MaBEL1, and MaCCCH33‐Like2, further highlight the intricate transcriptional networks orchestrating banana fruit maturation (Fan et al., 2018b; Song et al., 2023, 2024). The degradation of starch and accumulation of sugars during ripening is governed by a diverse array of metabolic genes including *MaGWD1*, *MaLSF2*, *MaBAM* family genes, *MaAMY3*, *MaISA3*, and *MaSPS*. These genes are involved in the conversion of complex carbohydrates into simpler sugars, improving fruit sweetness and palatability (Jourda et al., 2016; Miao et al., 2016; Fan et al., 2018b; Wang et al., 2019; Liu et al., 2022). Transcription factors, such as MabHLH6, MADS‐box, MaMYB16L, and MaBEL1, play vital roles in regulating starch degradation (Xiao et al., 2018; Jiang et al., 2021; Liu et al., 2021; Song et al., 2023). Genes like *MaDXR1*, *MaPDS1*, *MaZDS1*, and *MaSPL16* have been shown to mediate carotenoid biosynthesis under high‐temperature conditions, contributing to fruit coloration and nutritional enhancement (Zhu et al., 2023). Moreover, MaNAC029 transcriptionally activates genes involved in aroma biosynthesis, adding sensory quality to the ripening fruit (Wei et al., 2023).

Banana plants are highly susceptible to a range of biotic stressors, specifically FW, that lead to substantial yield losses and diminished fruit quality. Several disease resistance genes have been characterized, including *2OGD*, *PR*, *MaLYK1*, *LRR‐RLP*, *MYB36*, and *Macma4_11_g19760*, which function in pathogen recognition and downstream defense activation (Zhang et al., 2019a; Álvarez‐López et al., 2022; Chelliah et al., 2023; Tian et al., 2023; Li et al., 2023b Anuradha et al., 2024). Overexpression of genes such as *Ced9*, *RGA2*, and *MpbHLH* has been associated with enhanced resistance to FW. Conversely, transgenic plants expressing *MaWRKY24* have shown increased susceptibility to *Foc* TR4, underscoring the complex role of certain TFs in defense regulation (Liu et al., 2019). Similarly, banana plantations are highly sensitive to harsh environmental conditions, including heat, cold, drought, heavy rainfall, salinity, and strong winds, all of which can lead to substantial yield reductions (Chaudhari et al., 2023). Abiotic stress tolerance is equally imperative for maintaining banana productivity under changing climatic conditions. Stress‐responsive genes, including *MaAQP*, *MaAGPase*, *MaMPK5*, *MAPKKK*, *MAPKK*, *MaCCO*, *MaU‐box*, *LysM*, *SRO*, *LOX*, *Expansin*, *MKK2* family genes, *MaROP5g*, *MaCCS*, *MaGHMP*, and *WOX*, have demonstrated strong activation under various abiotic stress conditions, supporting their role in broad‐spectrum stress adaptation (Feng et al., 2016; Hu et al., 2017; Miao et al., 2018; Zhang et al., 2019a; Tak et al., 2021; Backiyarani et al., 2022a, 2022b;[Bibr jipb70025-bib-0011] Chaudhary et al., 2022; Chaturvedi et al., 2023; Fan et al., 2023; Zeng et al., 2023; Wang et al., 2023a, 2023b; Anuradha et al., 2024). Moreover, the overexpression of *MaPSY*, *MusaDHN‐1*, *MusaWRKY71*, *MusaNAC042*, *MusaNAC29*, *MaPIP2‐7*, and *MaDREB1F* has also resulted in significantly improved tolerance to abiotic stressors (Shekhawat et al., 2012; Kaur et al., 2017; Jangale et al., 2019; Tak et al., 2021; Chaudhari et al., 2023; Negi et al., 2023; Xu et al., 2023). Collectively, these advances in banana functional genomics not only deepen our comprehension of trait regulation and environmental response but also provide a valuable foundation for future genetic improvement efforts aimed at improving yield, quality, and resilience in one of the world's most important staple crops.

## GENOME ENGINEERING APPROACHES FOR BANANA IMPROVEMENT

The institution of genetic engineering has remarkably transformed plant breeding, empowering the precise integration of genes into crops that might not exist naturally in their gene pool. By deploying this approach, genetically engineered crops expressing traits, like biotic and abiotic resistance, raised nutritional content, and prolonged shelf life, have been designed. Similarly, genetic engineering methods have also been used extensively in developing resistant banana cvs. with improved nutritional attributes, effectively countering biotic and abiotic threats, more especially when no host resistance is spotted in the banana germplasm. These initiatives have assisted in the integration of beneficial traits, effectively evading the extended generation time, polyploidy, and sterility challenges prevalent in myriad banana vars. Moreover, this approach holds promise in mitigating post‐harvest crop losses, improving food security, and enabling biofortification of nutraceuticals. While no genetically modified bananas or plantains have been released for commercial use, their cultivation must be acknowledged and promoted through rigorous scientific understanding and risk assessment investigations. This section explains the genetic engineering approaches used for investigating complex traits in bananas.

*Cisgenic/intragenic and transgenic recombination* approaches have emerged as a response to the continuous advancements in plant genetic engineering, addressing concerns about cross‐species and cross‐kingdom gene utilization (Schouten et al., 2006; Rommens et al., 2007). It is widely believed that genetic modifications using genes from within the same sp. improve consumer acceptance of genetically engineered crops (Lusk and Rozan, 2006). Two prominent examples of cisgenic/intragenic recombination in bananas involve Golden Bananas—enriched with pro‐vitamin A—which integrated a gene (*MtPsy2a*, phytoene synthase 2a) from “Asupina” (a banana cv. with high carotenoid content) (Paul et al., 2017, 2018), and FW‐resistant bananas, which incorporated an R gene analog 2 (*RGA2*) from a TR4‐resistant wild banana (Mam) (Peraza‐Echeverria et al., 2009). Researchers further transferred the *RGA2* gene into the Cavendish banana and confirmed its effectiveness in improving resistance against FW through 3 years of field trials and gene expression analyses (Dale et al., 2017a). In February 2024, the genetically modified Cavendish var. (QCAV‐4) containing the *RGA2* gene from Mam was approved for cultivation, marking the world's first approved transgenic banana (https://www.newscientist.com/article/2417568-genetically-modified-banana-approved-by-regulators-for-first-time/).

**Figure 4:**
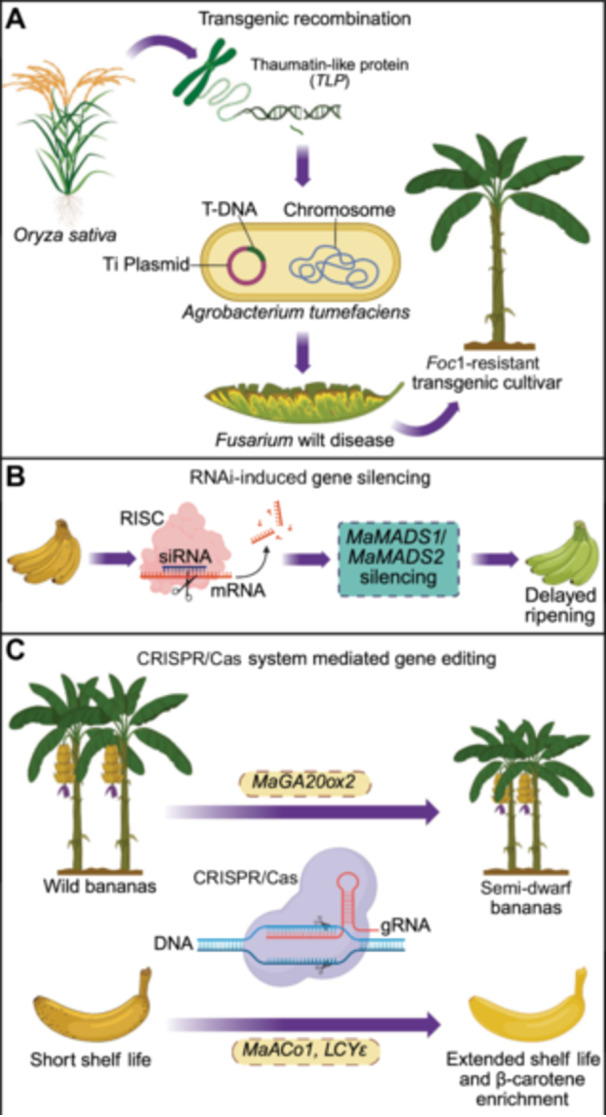
Genome engineering approaches for banana improvement and breeding through the manipulation of multiple genes **(A)** Introducing the *TLP* gene from rice into bananas to create a *Foc1*‐resistant transgenic cultivar (Mahdavi et al., 2012). **(B)** RNAi‐induced gene silencing to produce bananas with delayed ripening (Elitzur et al., 2016). (The genes modified for genetic improvement are indicated.) **(C)** Gene editing using the CRISPR/Cas9 technique to design semi‐dwarf bananas (Shao et al., 2020), and bananas with extended shelf life(Hu et al., 2021) and β‐carotene enrichment (Kaur et al., 2020). CRISPR/Cas9, Clustered Regularly Interspaced Short Palindromic Repeats/CRISPR‐associated protein 9; *Foc1*, *Fusarium oxysporum* f. sp. *Cubense* race 1; gRNA, guide RNA; RNAi, RNA interference; RISC, RNA‐induced silencing complex; T‐DNA, transfer DNA.

Implementation of transgenics in bananas is propitious because of their vegetative mode of propagation and parthenocarpic fruit development. Consequently, the risk of transgene spread to wild relatives is attenuated, ensuring the preservation of bananas' biodiversity (Waltz, 2014). Some genes for FW resistance from different spp. have been introduced into bananas through a transgenic approach. These may include genes encoding magainin analog (*MSI‐99*) from *Xenopus laevis* (Chakrabarti et al., 2003), human lysozyme from *Homo sapiens* (Pei et al., 2005), anti‐apoptosis genes (*BCL‐XL*, *BCL‐2*, *CED‐9*) from *H. sapiens* and *Caenorhabditis elegans* (Paul et al., 2011), *thaumatin*‐like protein (*tlp*) from *Oryza sativa* (Mahdavi et al., 2012), endo β‐1,3‐glucanase from *Glycine max* (Maziah et al., 2007a), rice chitinase from *O. sativa* (Maziah et al., 2007b), plant ferredoxin like protein (*pflp*) from *Capsicum annuum* (Yip et al., 2011), *Petunia* floral defensins *PhDEF1;2* from *Petunia hybrid* (Ghag et al., 2012), antimicrobial peptide (*Ace‐AMP1*) from *Allium cepa* (Mohandas et al., 2013), *Stellaria media* defensin (*Sm‐AMP‐D1*) from *Stellaria media* (Ghag et al., 2014a), and antimicrobial peptides *Ace‐AMP1* and *Ca‐pflp* from *A. cepa* and *C. annuum*, respectively (Sunisha et al., 2020). The generality of these experiments is confined to greenhouse conditions; hence, the practical effectiveness of these genes to engineer *Foc* resistance in bananas still needs to be assessed under field conditions (Figure 4A).

Banana plantations are menaced by other pathogens, including bacteria, viruses, and nematodes. Bananas with resistance toward banana bacterial wilt (BBW) caused by *Xanthomonas campestris* pv. *musacearum* (*X. campestris*) have been genetically engineered. Genes encoding hypersensitive response‐assisting protein (*HRAP*) (Tripathi et al., 2010, 2014, 2022) and *pflp* (Namukwaya et al., 2012) from *C. annuum* and *XA21* pattern recognition receptor from *O. sativa* (Tripathi et al., 2014) have been introduced in these bananas. Furthermore, transgenic bananas resistant to the pathogenic nematode *Radopholus similis* have been engineered using the *cystatin* gene from *O*. s*ativa* and *Zea mays* (Table 6) (Atkinson et al., 2004; Roderick et al., 2012).

**Table 6:** Application of genetic engineering for banana improvement

Targeted genes	Source of gene	Banana cultivar	Modified traits	Genetic Engineering Approach	References
*MSI‐99*	*Xenopus laevis*	Rasthali	*Foc1* resistance	Transgenic	Chakrabarti et al., 2003
*Human Lysozyme*	*Homo sapiens*	Taijiao	*Foc1* resistance	Transgenic	Pei et al., 2005
*Endo β‐1,3‐glucanase*	*Glycine max*	Rasthali	*Foc1* resistance	Transgenic	Maziah et al., 2007a
*REP*	Brazilian	Brazilian	BBTV resistance	RNAi	Borth et al., 2011
*BCL‐XL* and *CED‐9*	*H. sapiens*, *Caenorhabditis elegans*	Lady Finger	*Foc1* resistance	Transgenic	Paul et al., 2011
*PFLP*	*Capsicum annuum*	Gros Michel	*Foc1* resistance	Transgenic	Yip et al., 2011
*ThEn‐42*, (*StSy*), and Cu, *Zn‐SOD*	*Trichoderma harzianum*, *Vitis vinifera* and *Solanum lycopersicum*	Grand Naine	Improved tolerance toward fungal disease	Transgenic	Vishnevetsky et al., 2011
*PhDEF1; 2*	*Petunia hybrid*	Rasthali	*Foc1* resistance	Transgenic	Ghag et al., 2012
*TLP*	*Oryza sativa*	Nangka	*Foc1* resistance	Transgenic	Mahdavi et al., 2012
*CHIT42*	*Trichoderma harzianum*	Furenzhi	*Foc1* resistance	Transgenic	Hu et al., 2013
*Ace‐AMP1*	*Allium cepa*	Rasthali	*Foc1* resistance	Transgenic	Mohandas et al., 2013
*Velvet* and *FTF1*	Rasthali	Rasthali	*Foc1* resistance	RNAi	Ghag et al., 2014b
*MusaDAD1*, *MusaBAG1*, *MusaBI*	Rasthali	Rasthali	*Foc1* resistance	RNAi	Ghag et al., 2014c
*Sm‐AMP‐D1*	*Stellaria media*	Rasthali	*Foc1* resistance	Transgenic	Ghag et al., 2014a
*MaMADS1*, *MaMADS*2	Banana	‐	Delayed ripening	RNAi	Elitzur et al., 2016
*RGA2 and CED‐9*	*M. acuminate* *Caenorhabditis elegans*	Grand Naine	TR4 resistance	Cisgenic	Dale et al., 2017a
*MtPsy2a*	Asupina	Dwarf Cavendish	Biofortified pro‐vitamin A and Iron	Cisgenic	Paul et al., 2017
Banana cisgenes	Grand Naine	Grand Naine	Provitamin‐A enhancement and *Foc1* resistance	Cisgenic	Dale et al., 2017b
*RAS‐PDS*	Rasthali	Rasthali	Chlorophyll and carotenoid content reduction	CRISPR/Cas9	Kaur et al., 2018
*MaLYK1*	Williams	Williams	TR4 resistance	RNAi	
*Ace‐AMP1* + Ca‐*pflp*	*A. cepa* and *C. annuum*	Rasthali	*Foc1* resistance	Transgenic	Sunisha et al., 2020
*LCYε*	Grand Naine	Grand Naine	β‐carotene enrichment	CRISPR/Cas9	Kaur et al., 2020
*MaGA20ox2*	Gros Michel	Gros Michel	Dwarfism	CRISPR/Cas9	Shao et al., 2020
*MaACO1*	Brazilian	Brazilian	Reduced ethylene synthesis and extended shelf life	CRISPR/Cas9	Hu et al., 2021
*HRAP* and *PFLP*	*C. annuum*	Gonja Manjaya, Sukali Ndizi	BBW resistance	Transgenic	Tripathi et al., 2010; Namukwaya et al., 2012; Tripathi et al., 2014; Tripathi et al., 2022

Abbreviations: BBTV, Banana bunchy top virus; BBW, banana bacterial wilt; *Foc1*, *Fusarium oxysporum* f. sp. *Cubense* race 1; RNAi, RNA interference; TR4, Tropical race 4.

*RNA interference (RNAi)‐based gene silencing* is a nascent approach to genetically surpass the plant traits using antisense RNA, hairpin RNA, or double‐stranded RNA to suppress the expression of particular genes. This stratagem—often referred to as RNAi—entails the coherence of a small RNA to its complementary target messenger RNA, effectively suppressing the expression of a specific gene (Ratcliff et al., 1997). The focus of silencing can be a gene that naturally exists in the plant, like a receptor, or it can be specific genes found in pests or pathogens that pose a threat to the plant. Genetically modified bananas with resistance toward various pathogens have been successfully designed leveraging the RNAi approach. For instance, a cv. “Rasthali” resistant to FW was genetically engineered, possessing intron‐hairpin RNAs that targeted two pivotal gene sequences, that is, *Velvet* and *Fusarium* TF (*FTF1*), in the *Fusarium* pathosystem (Ghag et al., 2014b). Moreover, scrutinizing the biological function of lysin motif‐containing receptor‐like kinase 1 of *M. acuminata* (*MaLYK1*) in response to TR4 in cv. “Williams” expounded its pre‐eminent role in monitoring pathogenic and symbiotic signals (Zhang et al., 2019a).

Bananas are also vulnerable to some viruses such as BSV, BBMV and BBTV. An RNAi approach has expedited the detection of viral genes encoding virulence‐associated proteins from the point of infection and then effectively inhibited their systematic transmission by targeting these genes (Ibrahim and Aragão, 2015). Some reports illustrating the development of genetically engineered bananas using RNAi included RNAi targeted genes encoding viral replicase‐associated protein (Rep) (Borth et al., 2011), BBTV‐G‐CP (Ismail et al., 2011), and Rep and ProRep (Shekhawat et al., 2012) against BBTV infection. Moreover, genetically engineered bananas with the reduced expression of *MaMADS1* or *MaMADS2* (ripening genes MADS‐box) have been designed using RNAi technology and the altered plants rendered delayed ripening and an extended shelf life (Figure 4B) (Elitzur et al., 2016).

*Clustered Regularly Interspaced Short Palindromic Repeats/CRISPR‐associated protein 9* (*CRISPR/Cas9*) *system‐mediated GE* has appeared as a potent tool for discerning gene function and improve crop breeding techniques (Feng et al., 2013; Lu et al., 2017). This approach has epitomized remarkable prospects in improving banana traits, such as the inactivation of endogenous BSV in the “Gonja Manjaya” plantains by mutating *ORF1*, *ORF2*, and *ORF3* sequences of BSV to inhibit their transcription or translation into functional viral proteins (Tripathi et al., 2019). Additionally, the system successfully mutated the phytoene desaturase (*RAS‐PDS*) gene from the carotenoid biosynthetic pathway (Kaur et al., 2018; Naim et al., 2018). Moreover, the *MaGA20ox2* gene‐modified semi‐dwarf phenotype was designed by leveraging CRISPR/Cas9 in the “Gros Michel” cv., featuring lower levels of bioactive gibberellins than the natural cv. (Shao et al., 2020). The CRISPR/Cas9 editing system holds the potential to improve fruit quality traits. For instance, this system was deployed to target lycopene epsilon‐cyclase (*LCYε*) (Kaur et al., 2020) and amino‐cyclopropane‐1‐carboxylate oxidase 1 (*MaACO1*) (Hu et al., 2021) genes; banana fruits with β‐carotene enrichment and reduced ethylene synthesis were developed leading to an extended shelf life during natural ripening (Figure 4C). Some research groups are currently engaged in increasing TR4 resistance in Cavendish bananas using CRISPR/Cas9, not only by repressing the expression of the TR4 susceptible gene but also by activating the inactive genes that provide resistance against TR4 (Dale et al., 2017a; Maxmen, 2019).

With access to complete banana genomes and robust tools, it is now feasible to develop extensive CRISPR‐mediated mutant libraries covering the entire genome. These libraries can be used for target gene discovery, functional gene analysis, and genetic enhancement in bananas. In the upcoming years, the combination of functional genomic studies and genetic transformation techniques holds promise for producing enhanced banana vars. with superior disease resistance and high yield, thus improving agricultural practices.

## CONCLUDING REMARKS AND FUTURE PROSPECTS

Advancements in banana breeding have been scarce due to genetic complexity, limited breeding methods, low fertility in female parents, sterility, and parthenocarpy. A range of ploidy levels—from diploid to tetraploid—and limited germplasm further complicate the situation. Constricted genetic diversity escalates banana's vulnerability to climate change and environmental challenges. To summarize these disputes, the integration of omic technologies and genome design breeding is a dire need towards develop superior banana cvs. In this context, we have described the existing gaps in banana research and proposed potential perspectives and schemes to propel the field forward.

Although reference genomes have become available for *Musa* species, a steady upgradation of these reference genomes is a prerequisite to improving their quality and representativeness. Attaining a complete, T2T haplotype‐resolved, and entirely accurate assembly can be considered the ultimate goal in the winding journey to improve the quality of the banana genome assemblies (Huang et al., 2023). This will approve the identification of unique genes and structural diversity in the regions containing “dark matter,” including centromeres and large duplicated segments (Gaut et al., 2018; Zhou et al., 2019; Liu et al., 2024). Meanwhile, similar to other clonal crops such as potato (Cheng et al., 2025) and grapevine (Peng et al., 2025; Xiao et al., 2025), heterozygosity in the banana genome makes it difficult to resolve due to divergence in subgenomes. To develop an intensive profile to unravel the genetic heterozygosity in banana genomes, it is necessary to generate haplotype‐resolved reference genomes. As a case in point, recent studies on the T2T and haplotype‐resolved genomes (Huang et al., 2023; Liu et al., 2023b; Xie et al., 2023) have provided insight into the uneven evolution of subgenomes and their role in disease resistance. In the future, deploying NGS to generate phased assemblies will speed up the detection of substantial SVs, including large inversions, within polyploid banana genomes. Apart from the nuclear genome, organellar genomes, that is, chloroplast and mitochondrial genomes, also have indispensable genetic elements (Wang et al., 2022; Wang and Wang, 2023). However, these extranuclear genomes are often neglected in genomic research, leading to the absence of organellar genomes in pretty much all of the published banana genome assemblies. We propose creating finished banana genomes that meet the 3C (contiguity, completeness, and correctness) criteria while also delving into organellar genomes and heterozygosity.

Genetic studies have shown that the wild resources have been augmented with domestication alleles, which are infrequent and scattered among modern landraces (Huang et al., 2012; Gaut et al., 2018). Domestication and modern breeding are evolutionary processes designed to identify beneficial and multiple target alleles (Meyer and Purugganan, 2013; Long et al., 2024). Fast‐tracking mining of beneficial alleles within banana cvs. and their wild relatives is paramount for banana improvement. Pangenomics or GWAS should be used to pinpoint the superior genes/alleles associated with traits of interest in bananas. Pangenomes have primarily concentrated on diverse cultivated variants of a single crop species, limiting their representation of the broader germplasm diversity (Zhou et al., 2022; Liu et al., 2024). These pangenomes, predominantly, can be regarded as sub‐pangenomes within the genus because they focus on a single species. The genus *Musa*, however, encompasses various spp. and genomes. To achieve a profound perception, we propose a shift toward a genus‐level pangenome for bananas. Owing to the strides in cost‐effective NGS technologies, we advocate a more inclusive approach, super‐pangenome (Li et al., 2023a). We surmise that within genus *Musa*, beneficial genes like R genes would have been transferred from one sp. to another by interspecific or intraspecific hybridization. We propose to construct at least one novel assembly for each *Musa* sp. as it minimizes prejudice in mapping sequencing data from diverse accessions of other species. The construction of a super‐pangenome is pivotal for an intensive exploration of both dispensable and species‐specific genes. Simultaneously, SV‐based GWAS should be deployed to detect known and uncatalogued SVs, that is, PAVs and copy number variations (CNVs), more especially PAVs associated with environmental adaptation, domestication, and breeding (Tan et al., 2012; Jin et al., 2023; Liu et al., 2024). These will be paramount findings for breeders, as calling PAVs across wild and domesticated lines would assist in retracing the beneficial alleles that may have been lost through genetic drift during breeding bottlenecks. These genetic variants will further serve as markers for GS, enabling the integration of desirable traits such as *Foc‐*related R genes found in wild banana species, into cultivated bananas.

Artificial intelligence (AI) is proving to be a robust tool in the sphere of crop genetics and breeding, endowing accurate phenotyping and multi‐omics data mining (Beans, 2020). As for developing new banana vars. through genome editing, an integral measure is the high‐throughput screening of these modified vars. to produce the desired traits. AI can be used to attain effective selection of extensive banana breeding populations, tailored to specific environmental conditions and stressors. To illustrate, unmanned aerial vehicles (UAVs), field scanning platforms, phenotyping towers, and spectral satellite imaging will provide breeders with broad phenotypic data, allowing efficient and thorough categorization of banana vars. based on their traits. Further than its contributions to phenotyping, AI is now instrumental in large‐scale genetic analysis, in which ML techniques can be applied to mine genetic information more effectively than ever before (Yang et al., 2023). By leveraging advanced algorithms, breeders can now prioritize candidate genes related to disease resistance, yield, and nutritional quality. As ML techniques progress and become more accessible, they will play a pivotal role in shaping the future of banana genomics, facilitating efficient and precise breeding techniques for improved banana productivity and sustainability.

**Figure 5:**
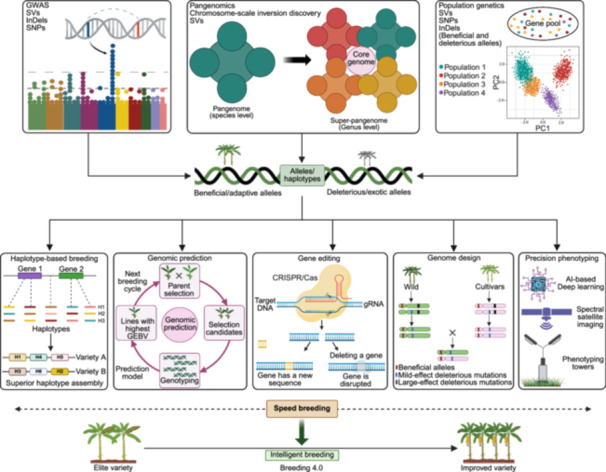
A sneak peek at the future Ascertaining beneficial and deleterious alleles within banana genetic resources can be expedited by using pangenomics, GWAS, and population genetics analyses. Novel adaptive or exotic alleles undergo additional steps for the efficient, accurate, and specific modification of important traits. Gene editing and genome design are powerful techniques that can serve to retain beneficial alleles while removing deleterious mutations in bananas (e.g., disease resistance). Haplotype‐based breeding relies on a specific group of important genes, while breeding techniques like genomic selection are expected to mitigate the genetic diversity within a breeding program over time. Advancements occurring concomitantly in image and sensor technologies will enable the acquisition of highly accurate phenotyping data. Integrating these tools with AI‐based approaches leads to intelligent breeding—referred to as Breeding 4.0—which unifies the advanced genomic technologies, high‐throughput phenotyping, and data‐driven computational approaches to accelerate and optimize crop development. This holistic strategy will facilitate the creation of superior banana cultivars critical for future food security. CRISPR/Cas9, Clustered Regularly Interspaced Short Palindromic Repeats/CRISPR‐associated protein 9; GEBV, Genomic estimated breeding value; gRNA, guide RNA; GWAS, Genome‐Wide Association Studies; InDels, Insertions and deletions; SNP, Single nucleotide polymorphism; SV, Structural variations.

With the intent of genomic engineering, de novo domestication of wild bananas can be implemented through simultaneous manipulation of multiple genes, as demonstrated in rice (Yu et al., 2021). Gene editing and genome design enable the meticulous modification of crucial domestication‐related genes. The innovative “prime editing” within CRISPR/Cas9 offers versatile editing capabilities, advancing banana improvement endeavors (Anzalone et al., 2019). Thus, concatenating GE and genome design with pangenomics and GWAS will provide an alternative route to engineer designer bananas, along with other widely used breeding technologies (e.g., deep‐GS: Abdollahi‐Arpanahi et al., 2020; haplotype‐based breeding: Brinton et al., 2020; and speed breeding: Alahmad et al., 2018). With the integration of modern genomics, phenomics, GE, and synthetic biology, combined with AI to form a Big Data‐driven, AI‐supported decision‐making pipeline, we can now enter the breeding 4.0 (intelligent breeding) era for bananas (Figure 5). To set foot on breeding 4.0 in bananas, we need to learn from tomato, potato, and rice breeding (Li et al., 2018; Zsögön et al., 2018; Yu et al., 2021; Zhang et al., 2021b). In our vision, it will be imperative to discover and remodel the key genes controlling *Foc* resistance from wild relatives into cultivated bananas, leveraging accelerated genome design breeding. The combination of multi‐omics and accelerated breeding will drive scientific breakthroughs in banana research, supporting global efforts to address food security challenges in the coming years.

## CONFLICTS OF INTEREST

The authors declare that they have no conflicts of interest.

## AUTHOR CONTRIBUTIONS

Y.Z. planned and designed the review. R.A. and T.R. wrote the initial draft of the manuscript. R.A., T.H., C.L., Z.J., H.‐R.H., and P.L. made the tables and drew the images. Y.Z., R.A., T.R., B.A., Z.L., W.Z., W.W., X.‐J.G., and J.X. edited and revised the review. All authors contributed to the proofreading of the manuscript.
